# Marine Caves of the Mediterranean Sea: A Sponge Biodiversity Reservoir within a Biodiversity Hotspot

**DOI:** 10.1371/journal.pone.0039873

**Published:** 2012-07-11

**Authors:** Vasilis Gerovasileiou, Eleni Voultsiadou

**Affiliations:** Department of Zoology, School of Biology, Aristotle University, Thessaloniki, Greece; Hawaii Pacific University, United States of America

## Abstract

Marine caves are widely acknowledged for their unique biodiversity and constitute a typical feature of the Mediterranean coastline. Herein an attempt was made to evaluate the ecological significance of this particular ecosystem in the Mediterranean Sea, which is considered a biodiversity hotspot. This was accomplished by using Porifera, which dominate the rocky sublittoral substrata, as a reference group in a meta-analytical approach, combining primary research data from the Aegean Sea (eastern Mediterranean) with data derived from the literature. In total 311 species from all poriferan classes were recorded, representing 45.7% of the Mediterranean Porifera. Demospongiae and Homoscleromorpha are highly represented in marine caves at the family (88%), generic (70%), and species level (47.5%), the latter being the most favored group along with Dictyoceratida and Lithistida. Several rare and cave-exclusive species were reported from only one or few caves, indicating the fragmentation and peculiarity of this unique ecosystem. Species richness and phylogenetic diversity varied among Mediterranean areas; the former was positively correlated with research effort, being higher in the northern Mediterranean, while the latter was generally higher in caves than in the overall sponge assemblages of each area. Resemblance analysis among areas revealed that cavernicolous sponge assemblages followed a pattern quite similar to that of the overall Mediterranean assemblages. The same pattern was exhibited by the zoogeographic affinities of cave sponges: species with Atlanto-Mediterranean distribution and Mediterranean endemics prevailed (more than 40% each), 70% of them having warm-water affinities, since most caves were studied in shallow waters. According to our findings, Mediterranean marine caves appear to be important sponge biodiversity reservoirs of high representativeness and great scientific interest, deserving further detailed study and protection.

## Introduction

Coastal marine hard substrata have a great ecological, scientific, and economic value due to their high structural complexity, which supports rich marine communities. A significant component of the rocky bottoms are carbonate rocks, where karstic processes have resulted in the formation of marine caves in many coastal marine areas of the world. This geomorphology is very prominent along the coastline of the Mediterranean Sea, more than half (54%) of which is rocky [1], covered mainly with limestone, this being one of the defining characteristics of the Mediterranean environment [2]. The climatic features along with the geological history of this region have provided its coastal rocky areas with a substantial number of more or less complex marine cave systems, including submarine, semi-submerged (also found in literature as littoral or superficial), as well as anchihaline caves, as defined in Stock *et al*. [3].

Marine caves are acknowledged for their rich biodiversity and in several instances are an integral part of hotspot areas [4]. Although they constitute a relatively discrete, difficult to approach environment, evidence on impacts of human activities [5] and warming temperatures attributed to climate change [6], [7] emphasizes the need to evaluate and protect their biodiversity. This becomes more urgent in the Mediterranean Sea, which is a recognized hotspot from both the biodiversity and the socio-economic point of view, since it i) hosts 7% of the world’s marine biodiversity (with only 0.82% of the oceans’ surface), ii) supports a large number of endemic species, and iii) suffers intense and variable anthropogenic pressure predicted to increase in the future [8]. The high endemism of the Mediterranean Sea provided strong motivation for the development of a coastal network of Marine Protected Areas (MPAs), but the current system of protection is not yet representative, since several areas and habitats are underrepresented [9], including the cave habitat.

In contrast to their terrestrial equivalent, the biology of marine caves had remained almost totally unexplored until the middle of the last century. The technological advances and development of skin and SCUBA diving techniques and equipment (i.e. Aqua-Lung open-circuit unit by Emile Gagnan and Jacques-Yves Cousteau in 1942–1943) triggered their investigation [10]–[12]. The early studies focused primarily on their faunal composition and community structure [13]–[15]. Riedl [12] gave a first census of Mediterranean marine cave diversity, recording 905 species. Information was subsequently added regarding the spatial [16] and temporal variability of cave-dwelling assemblages [17], [18].

The vast majority of the species recorded in the marine cave environment have been characterized as stygophilic (not specialized for subterranean life, found in other similar habitats as well) or stygoxenes (sheltering in caves but feeding outside). However, some caves, especially of the anchihaline type, host a rich and diverse, endemic, stygobiotic (cave-exclusive) fauna [19]. The anchihaline cavernicolous species usually present a very disjunct geographic distribution and thus they have been widely investigated in biogeographic studies [20]–[23]. On the contrary, the significance of submarine cave fauna from this point of view is underestimated, despite the fact that their study has revealed rare or previously unrecorded cryptic species with bathyal affinities [24]–[26], relict species, and living fossils [27]–[29]. Although the general significance of marine caves is underlined in the numerous relevant studies, there is an apparent lack of a comprehensive quantitative approach on the representativeness of this specific habitat and its biodiversity for the ecosystem of the Mediterranean Sea.

Porifera are widely recognized as the dominant taxon in marine cave communities, in terms of species richness, spatial coverage, and biomass [30], [31]. Sponges, as mostly sciaphilic animals [32], are favored by the elimination of light and take advantage of the disappearance of space competing macroalgae towards the cave interior, turning this semi-dark environment into a real ‘sponge realm’. Cave sponge research has focused on semi-submerged and submarine caves of the Mediterranean Sea [33]–[36] and coral reef caves of the West Indian Ocean [37], while little attention has been paid to marine cave sponges from temperate regions [38]. A rapid assessment of the research effort concerning the world marine cave sponge biota through ISI Web of Science, by using the keywords ‘sponge’ and ‘cave’, revealed 97 relevant studies, out of which 63 (64.9%) were undertaken in the Mediterranean Sea, while only 15 (15.5%) have taken place in the Atlantic Ocean and 19 (19.6%) in the Indo-Pacific coasts.

Besides being dominant cave dwellers, sponges are a prominent invertebrate group in hard-bottom benthic communities of the Mediterranean Sea. They develop complex associations with other organisms, supporting diverse microbial and macrofaunal communities [39]; they act as ecosystem engineers, increasing habitat complexity [40], which is very important in space-limited environments, such as submarine caves. Due to their sessile life habit and limited ability for dispersal, they have been used in the investigation of zoogeographical patterns and affinities of the Atlanto-Mediterranean [41]–[45] and other regions [46], [47]. Considering the high taxonomic knowledge of sponges in the Mediterranean, they can be used as an appropriate study group, representative of cave species diversity and distribution.

An impediment towards this goal could be the fact that cave sponge communities are not homogenously studied across the Mediterranean. A plethora of marine caves have been explored in its north-western part, some of them intensively [48]–[50], while only scattered information exists, for example, on the sponges of the north-eastern part [51]. Thus, further research is considered necessary in order to have a sufficient estimation of cavernicolous sponge communities in the Aegean Sea, the northern part of which has proven to be a very prominent area within the Eastern Mediterranean, hosting a comparably rich sponge fauna [44], [52].

In the current study, apart from providing new data concerning sponge diversity from Eastern Mediterranean marine caves, Porifera are used as a reference group at a pan-Mediterranean scale for a meta-analysis aiming to evaluate the ecological significance of this particular habitat for the Mediterranean ecosystem by: i) examining through a quantitative approach whether marine caves constitute biodiversity reservoirs and centers of endemism, ii) estimating the diversity they support in comparison to that of the entire Mediterranean, and iii) investigating potential zoogeographical patterns among cave communities in the different subareas of the Mediterranean basin. The outcomes of this work aim to highlight the biological significance of this unique habitat for the Mediterranean region, point out regional gaps in the knowledge of cave communities, and provide a basis for integrating information into conservation planning.

## Methods

### Ethics Statement

This study has been approved by the Aristotle University of Thessaloniki and the Greek Ministry of Education, Lifelong Learning and Religious Affairs through the acceptance of a Ph.D. project proposal. No specific permissions were required for sampling locations/activities. The locations studied are not privately-owned or protected in any way. Sponge sampling was performed through non-destructive methods.

### Study Areas and Sponge Sampling

Qualitative sampling was performed in nine marine caves, four from the North and five from the South Aegean Sea: ‘Trypia Spilia’ (39°32′5.94′′ N, 24°58′′39.72′′ E) and ‘Ftelio’ (39°30′13.74′′ N, 24°58′16.44′′ E) caves in Agios Efstratios island; ‘Fará’ (38°58′11.64′ N, 26°28′39.54′′ E) and ‘Agios Vasilios’ (38°58′13.25′′ N, 26°32′30.46′′ E) caves in Lesvos island; Madhes (35°24′9.62′′ N, 25° 2′2.46′′ E), Alykes (35°25′3.19′′ N, 24°59′6.78′′ E) and Stavros (35°25′52.79′′ N, 24°58′20.67′′ E) caves in Crete; one in Andros Island (37°48′56.62′′ N, 24°58′43.24′′ E) and one at Vouliagmeni coast, Attica (37°47′58.13′′ N, 23°47′20.75′′ E). Sampling was performed by SCUBA diving. Sponge specimens and spicule preparations have been deposited in the Zoological Museum of the Department of Zoology, Aristotle University of Thessaloniki. The followed classification is in agreement with that proposed in Systema Porifera [53], the World Porifera Database (WPD) [54], the World Register of Marine Species (WoRMS) [55], and the Integrated Taxonomic Information System (http://www.itis.gov).

### Faunistic Data Collection and Taxonomic Updating

In order to obtain the entire available information on sponge assemblages reported from Mediterranean marine caves, all relevant literature was examined. Literature was primarily derived from an extensive base (mostly old publications) on Mediterranean marine biology kept in the Laboratory of Zoology (School of Biology, Aristotle University). This material was crosschecked and updated by keyword searching the ISI Web of Science and completed by examining in detail all references cited in the resulting articles. Eventually, this search yielded 256 publications, covering a time span of 63 years. Porifera proved to have been thoroughly studied in the marine cave environment since they appeared in more than half of the publications, with one third of them devoted exclusively to this specific group.

Literature data and new records from caves of the North and South Aegean Sea (current survey) were incorporated into a Microsoft Excel database along with spatial (area, depth) and ecological (cave zone) information. In order to evaluate the scientific research effort devoted to caves in the Mediterranean Sea, scientific studies were classified according to year of publication, region, and scientific approach (ecological/faunistic or systematic).

Regional data on cave sponges were used in order to identify potential biogeographical patterns among and within cave assemblages. In this assessment only the classes Homoscleromorpha and Demospongiae, making up 84% of the global poriferan diversity [56], were taken into account, since they appeared homogenously studied along the surveyed Mediterranean caves. The biogeographical division of the Mediterranean Sea was based on that proposed by Pansini and Longo [41] as modified by Voultsiadou [44] and included the following areas: Spanish coast (SC), French coast (FC), Ligurian Sea (LS), Tyrrhenian Sea (TS), Tunisian coast (TC), North Adriatic (AN), South Adriatic (AS), Ionian Sea (IS), North Aegean (NA), South Aegean (SA) and Levantine Basin (LB). Since relevant data for the Alboran Sea, Algerian and Egyptian coasts are scarce or totally missing, these areas were not included in the analyses.

Spearman’s rank correlation coefficient was calculated with IBM SPSS Statistics to investigate the relationship of species richness with the number of publications and number of caves studied in each area, as a non-parametric measure of statistical dependence.

The occurrence of sponge species in marine caves of the above areas was incorporated into a presence/absence matrix, including the total number of caves and the cave zones in which each species was recorded. For comparison purposes, a similar matrix was compiled for the overall Mediterranean species of the two examined classes by updating previous lists [41], [44] with records added thereafter [57]–[60]. With regard to species distribution in the different Mediterranean areas, new records from the North Aegean (present work), South Aegean (present work, [40], [61]), Levantine [61], Ionian [62], North and South Adriatic [50], and Tyrrhenian seas [63], [64], were taken into account. Since the species inventory was compiled from studies published in different years (1949 to the present) a taxonomic updating was considered necessary. This was accomplished by consulting relevant literature sources and cross-checking with the WPD and the WoRMS.

### Diversity and Similarity Analysis

Τo evaluate overall and regional diversity of the Mediterranean cave sponge assemblages, besides the commonly used species richness, two other indices, robust to variation in sampling effort and capturing the phylogenetic or taxonomic diversity, were applied to our data: average taxonomic distinctness (AvTD or Δ+) and variation in taxonomic distinctness (VarTD or Λ+) as defined by Clarke and Warwick, [65] and [66] respectively. The former calculates the average degree to which species in an area are taxonomically related to each other, i.e. the taxonomic breadth of the assemblage, with high values indicating a greatest amount of phylogenetic diversity, or “evolutionary history”. The latter reveals the evenness of the distribution of taxa across the taxonomic tree, with high values indicating that some taxa are overrepresented and others underrepresented by comparison to what we know for the species pool of the geographical region. In addition to the calculated values for each of these two indices, an estimation of the departure from expectation is depicted with confidence funnels, showing whether the diversity of an area is above or below simulated probability limits [67]. Both are ideal for comparisons of biodiversity over historic time or biogeographic space scales and have already been used in biogeographic studies regarding Porifera [44], [45]. For their calculation, in addition to the species matrices mentioned above, an aggregation file was constructed relating all taxa at the different taxonomic levels.

Faunal affinities among the different Mediterranean regions were estimated using the Bray-Curtis coefficient. Similarity matrices were constructed from the above mentioned presence/absence matrices and multivariate analyses, i.e. hierarchical clustering and multidimensional (MDS) scaling, were performed. The same procedure was followed for the assemblages of each cave zone separately for the areas, for which this kind of spatial information was available i.e. SC, FC, TS, AN, AS, and NA.

All the above analyses were performed using PRIMER v6 Package [68].

### Biogeographic Comparisons

In order to assess biogeographic affinities of cave sponge fauna, a classification of species based on their worldwide distribution and their distribution in the different temperature zones was made. Sponge species were assigned into five categories based on their geographical distribution (as this is presented in the WPD) namely Mediterranean endemics, East Atlanto-Mediterranean, Amphi Atlanto-Mediterranean, Indo-Mediterranean, and Cosmopolitan. The species with Atlanto-Mediterranean distribution were assigned according to their distribution in the main temperature zones (biogeographic regions), as these have been realigned by Briggs and Bowen [69], into three bioclimatic categories, cold, warm water, and eurythermic species. Mediterranean endemics were considered *a priori* as warm water species.

## Results

### New Data on Sponge Diversity of Aegean Sea Caves

As mentioned in the Introduction, the Aegean Sea was one of the less studied Mediterranean areas in terms of its submarine cave fauna. The current research in caves of the North and South Aegean revealed the presence of 80 sponge taxa, 61 of which were identified to the species level. Out of these species, 11 are new records for the fauna of the Aegean Sea and 3 new for the Eastern Mediterranean (see Table S1).

Most of the species found (58) are new records for the marine caves of the Aegean Sea, while three species (*Haliclona perlucida, Hyrtios collectrix, Protosuberites rugosus*) are reported for the first time as cave dwellers, the latter having deep sea affinities. Taking into account previous scattered information, the sponge fauna found so far in marine caves numbers totally 90 taxa (71 to the species level) in the North and 33 in the South Aegean.

### Sponge Diversity and Distribution in Mediterranean Marine Caves

#### Research effort overview

Concerning the Mediterranean cave sponge fauna, a detailed evaluation revealed that half of the studies focused exclusively on marine caves, while the remaining were more general works investigating other hard substrate sciaphilic habitats and including data on cave sponges.

Sponges of Mediterranean marine caves were surveyed in 133 publications (Text S1), 92% of which can be, according to their research approach, equally divided into ecological/faunistic and systematic ones. A minimum of 185 Mediterranean marine caves have been explored for their sponge fauna. Most of them (70%) were semi-submerged, or shallow marine caves with a maximum depth not exceeding 15 m. Only 25% included deeper sectors, with 12 marine caves reaching or surpassing the depth of 30 m and 3 having a maximum depth of 40 m. Bathymetric data are lacking for the remaining studied caves. Caves with deeper sectors were more numerous along the French coasts, constituting 56% of the caves explored in the area, and 30% of the Mediterranean deeper caves. In contrast, South Adriatic, Tyrrhenian, and Levantine caves were almost all shallow. Only four caves were anchihaline, located in the Spanish coasts (Cova des Coll, Mallorca), North and South Adriatic islands of Croatia (Ziva Voda and Medvjeda Spilja respectively), and Ionian coasts of Italy (Zinzulusa).

**Figure 1:**
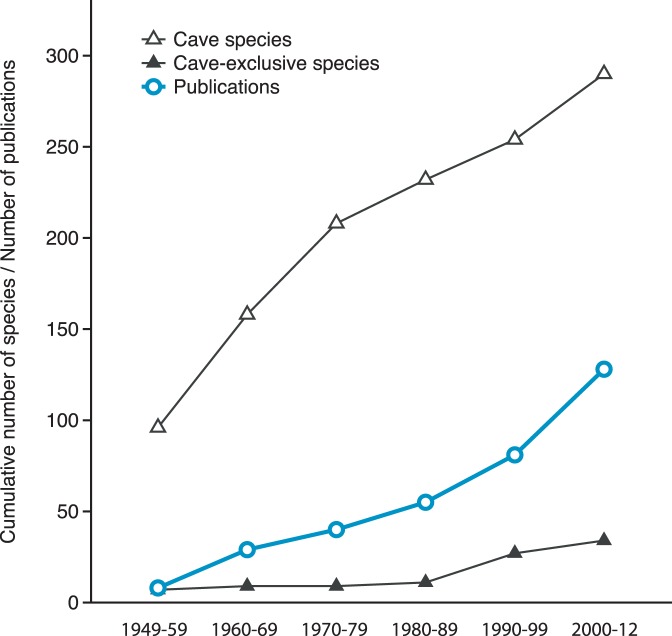
Sponge species recorded from Mediterranean marine caves through time, alongside with research effort.

**Figure 2:**
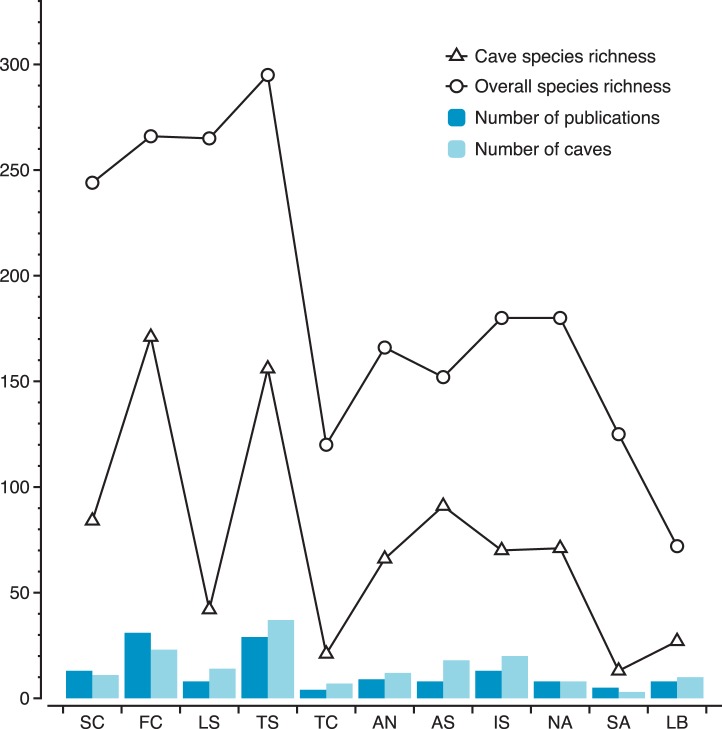
Research effort compared to sponge species richness in Mediterranean subareas. For abbreviations of Mediterranean areas see Methods.

The study of Mediterranean caves started in the fifties and has an upward trend over the years (around 4 papers per year, 2 of which include sponges). A similar trend is exhibited by the addition of new species to the cave-dwelling sponge fauna, as well as by the detection of cave-exclusive sponges (Fig. 1). The research effort (number of publications or number of caves explored) is not equal over the Mediterranean (Fig. 2). Some areas, such as the French and Tyrrhenian coasts, have been intensively studied, this evidenced in the former primarily by the big number of publications and in the latter by the plethora of the explored caves. In the Adriatic and Ionian Sea, which have received moderate research intensity, the number of caves surveyed was bigger than the number of studies conducted. Other areas, such as the coasts of Tunisia and South Aegean have received the least attention regarding their sponge cave fauna. The recent exploration of a small number of caves in the Levantine and the Aegean Sea has remarkably increased cave sponges richness known from these areas. In general, the number of cave species reported from each area was positively correlated with the number of publications and the number of caves explored (*r^2^* = 0.820 and 0.817 respectively, *P*<0.01, *n* = 11).

#### Overall diversity

The exhaustive study of the literature showed that, currently, 45.7% of the Mediterranean Porifera have been found in marine caves. In total, 311 species (270 Demospongiae, 20 Homoscleromorpha, 20 Calcarea, and 1 Hexactinellida) were recorded (Table S2), showing that all poriferan classes can be found in caves. However, considering the total Mediterranean Porifera species (588 Demospongiae, 22 Homoscleromorpha, 60 Calcarea, and 11 Hexactinellida), Homoscleromorpha is highly represented (91%) in the cave environment, Demospongiae and Calcarea by 46% and 33% respectively, while only 9% of the Mediterranean Hexactinellida were found in caves.

**Figure 3:**
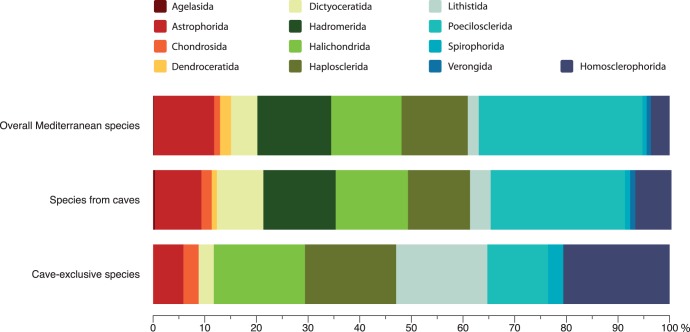
Order composition of cave and overall Mediterranean Demospongiae and Homoscleromorpha.

Focusing on Demospongiae and Homoscleromorpha, caves host all 13 orders. The general distribution pattern of species number in the above orders is similar in both the cave environment and the total Mediterranean habitats (Fig. 3). However, the former seems more favorable for the sponges of the orders Dictyoceratida, Lithistida, and Homosclerophorida, while Astrophorida and Poecilosclerida are underrepresented in cave assemblages in relation to the overall Mediterranean ones.

Examining cave sponge diversity at the family level, it should be noted that although a high percentage (88%) of the families have been found, Azoricidae, Desmacellidae, Desmacididae, Esperiopsidae, Latrunculidae, Niphatidae, Podospongiidae, and Stylocordylidae are missing. Finally, 70% of the Mediterranean genera and 47.5% of the species have been found up to date in marine caves. It should be mentioned that 97 taxa examined from different caves and areas remain identified only to the genus/family level and 9 [*Aka labyrinthica* (Hancock, 1849), *Darwinella australiensis* Carter, 1885, *Desmanthus incrustans* (Topsent, 1889), *Erylus expletus* Topsent 1927, *Eurypon viride* (Topsent, 1889), *Gelliodes luridus* (Lundbeck, 1902), *Lissodendoryx (Lissodendoryx) isodictyalis* (Carter, 1882), *Rhabderemia indica* Dendy, 1905 and *Rhabderemia minutula* (Carter, 1876)] are presently considered as invalid Mediterranean records.

**Figure 4:**
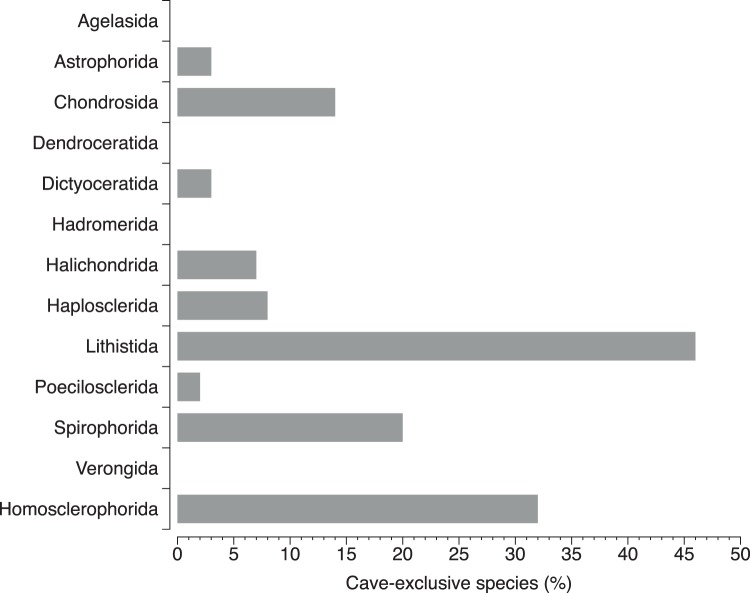
Percentages of cave-exclusive species in the Demospongiae and Homoscleromorpha orders.

Thirty-four species appear as cave-exclusive (Table S2), since they have been recorded only in marine caves, making up 11.7% of the Mediterranean cave Demospongiae and Homoscleromorpha fauna and 5.6% of the overall Mediterranean one. The majority of these cave-exclusive species have been found in only one cave, while several are rare species recorded from a small number of caves. Homosclerophorida is the dominant order of cave-exclusive sponges, followed by Lithistida, Halichondrida, and Haplosclerida (Fig. 3). Moreover, for some poriferan orders, such as Lithistida, Homosclerophorida, and Spirophorida, the cave environment is a very preferable habitat, since 46, 32, and 20% of their species have been found exclusively in caves (Fig. 4).

Finally, certain species with deep sea affinities have been recorded in the dark sections of caves, regardless of depth: the Demospongiae *Asbestopluma hypogea*, *Calthropella pathologica*, *Discodermia polymorpha*, *Eurypon clavatum*, *Hamacantha (Hamacantha) papillata*, *Merlia normani*, *Monocrepidium vermiculatum*, *Neoschrammeniella bowerbanki*, *Petromica (Petromica) grimaldii*, *Petrosia (Strongylophora) vansoesti*, *Protosuberites rugosus*, *Pseudotrachya hystrix*, *Rhizaxinella pyrifera*, *Spiroxya levispira*, *Thenea muricata*, *Thrombus abyssi*, and the hexactinellid *Oopsacas minuta*.

#### Regional diversity patterns

As mentioned above, sponge species richness in marine caves differed among the Mediterranean areas, mostly following the number of publications and caves explored (Fig. 2, Table S3). In most areas it ranged between 60 and 100 species, being notably high (>160 species) in the French and Tyrrhenian coasts, but low (<40) in the coasts of Tunisia, South Aegean, and the Levantine. This pattern does not fully agree with that of sponge species richness found in the above Mediterranean areas, when all habitat types are taken into consideration. On the Spanish and Ligurian coasts, which host a remarkable sponge fauna, a contrastingly low sponge species number has been reported from caves, while an opposite trend is observed for the coasts of South Adriatic, the North Aegean, and the Levantine.

**Figure 5:**
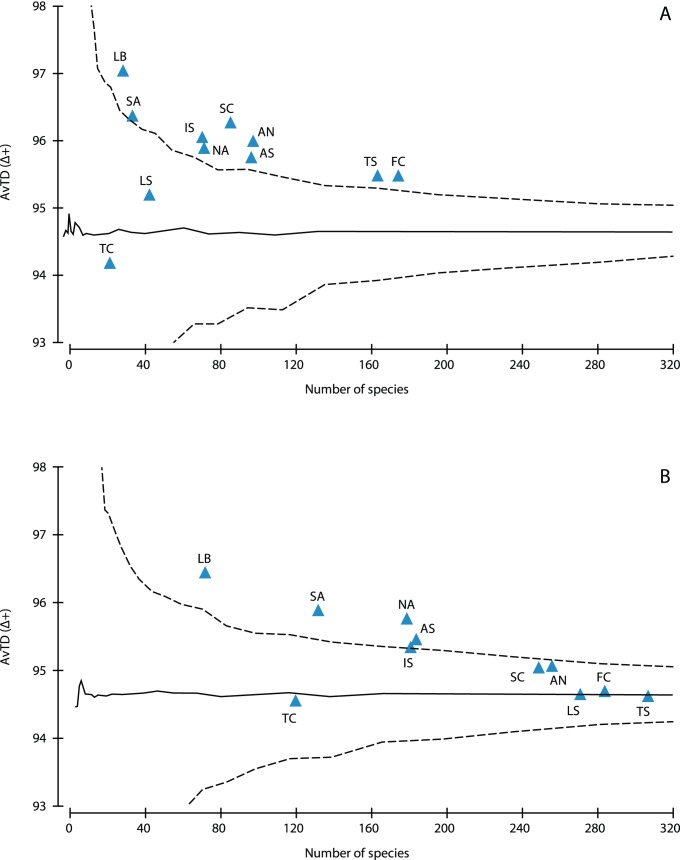
Average taxonomic distinctness (AvTD) for the Mediterranean subareas. (A) cave sponge fauna, (B) overall Mediterranean sponge fauna. The central line represents the mean value and the funnel curve shows the limits for its expected values. For abbreviations of Mediterranean areas see Methods.

**Figure 6:**
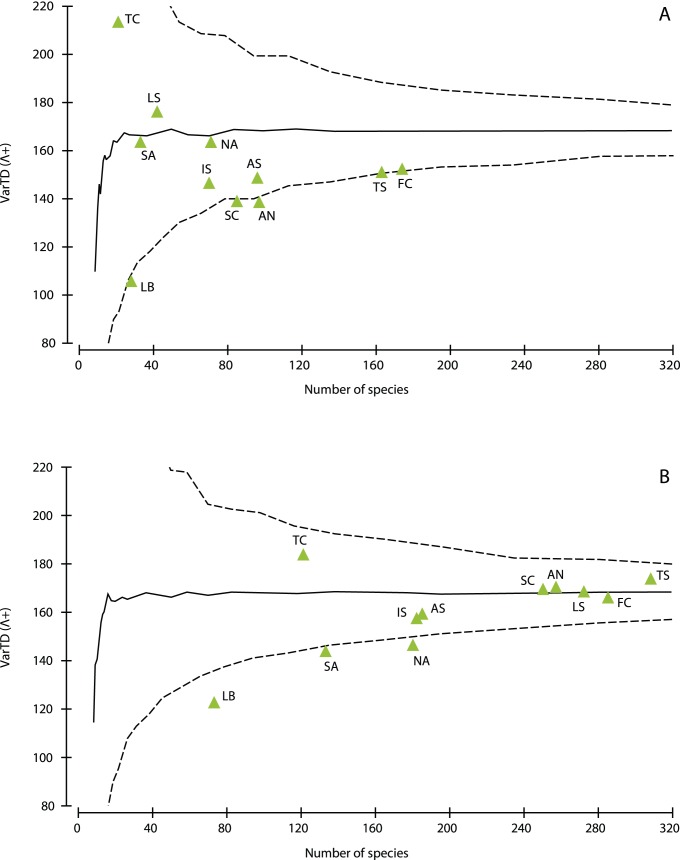
Variation in taxonomic distinctness (VarTD) for the Mediterranean subareas. (A) cave sponge fauna, (B) overall Mediterranean sponge fauna. The central line represents the mean value and the funnel curve shows the limits for its expected values. For abbreviations of Mediterranean areas see Methods.

Besides species richness, average taxonomic distinctness also varied in the Mediterranean subareas (Fig. 5A, Table S3). However, its values for cave assemblages from all areas were around (Tunisian coast and Ligurian Sea) or above the mean, even above the upper limit of expectation. A comparison of these values with the taxonomic distinctness of the same areas, when their overall sponge fauna was considered (Fig. 5B; Table S3), showed that the latter were more closely concentrated around the mean, with the North and South Aegean, and the Levantine being still above the upper limits of expectation. Variation in taxonomic distinctness for the cave sponge fauna (Fig. 6A) were generally inside the limits of expectation, with most of them below the expected mode, and for most areas they were lower than the corresponding values for the overall sponge fauna (Figure 6Β). Higher values of this biodiversity index were observed for the two Aegean regions in caves, as compared to the overall sponge assemblages; in the former case it had an average value, near the mean expected, while in the latter it was on the lower limits of expectation.

**Figure 7:**
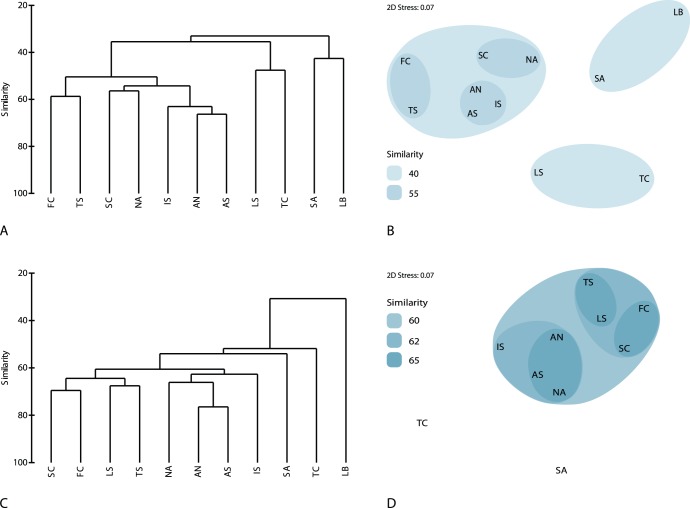
Resemblance of sponge assemblages in the Mediterranean subareas demonstrated in dendrograms and non-metric MDS plots. (A, B) cave assemblages, (C, D) overall Mediterranean assemblages. In the latter analysis, data for the Levantine Basin were omitted (see Methods). For abbreviations of Mediterranean areas see Methods.

Estimation of resemblance among the cave sponge fauna of the Mediterranean subareas (Fig. 7A, B) revealed three groups at a similarity level of 33%. One included all northern Mediterranean subareas (those of the western basin, Adriatic, Ionian and North Aegean Sea), except for the Ligurian Sea, which formed a second group with the Tunisian coast. Inside the former group, at 50% similarity, the French coasts and the Tyrrhenian Sea were separated from the other north Mediterranean areas (Spanish coast, North Aegean, North and South Adriatic, and Ionian), which were grouped together. The South Aegean formed a third group with the Levantine at a similarity level of 43%. A slightly different pattern was observed when all Mediterranean sponge species were treated together (Fig. 7C, D). The north-western Mediterranean areas (French, Spanish, Ligurian, and Tyrrhenian coasts) were grouped together as did the north-eastern areas (South and North Adriatic, Ionian and North Aegean) with a similarity of more than 60%. In this case, the Tunisian coast and South Aegean were separated from all other areas, while the Levantine was considerably dissimilar and thus not included in the analysis.

Few sponges appeared rather widely spread in marine caves, with only 4 of them, the common species *Petrosia ficiformis, Ircinia variabilis, Agelas oroides,* and *Spirastrella cunctatrix,* having been found in 33–36% of the Mediterranean caves explored. On the contrary, 67% of the species were recorded in less than 5 caves (34.5% in only one cave). Among the latter belong some rare species, such as *Cinachyrella levantinensis*, *Coscinoderma sporadense*, *Hemiasterella aristoteliana*, *Didiscus spinoxeatus,* and *Polymastia harmelini*, with limited distribution in only one Mediterranean area (see Table S2).

Sponge species richness varied among the different caves studied (Table S4). The two caves richest in sponges (Cave of Figuier and Cave of Endoume, both with more than 70 species) are located on the French coast and are among the most thoroughly studied ones. The four North Aegean caves studied here for the first time were ranked among the first 40 of the total 185 Mediterranean caves, while Fará and Agios Vasilios are among the 20 richest caves.

**Figure 8:**
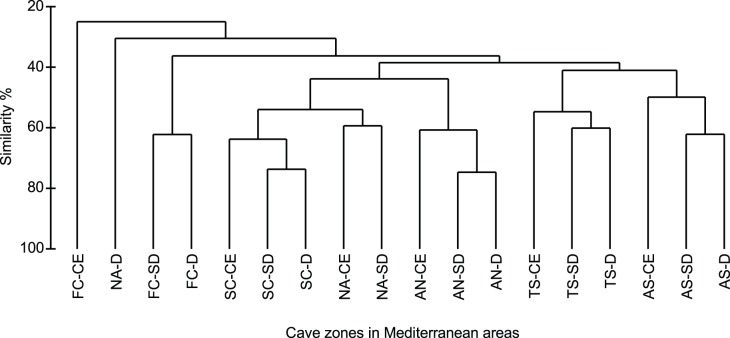
Resemblance of sponge assemblages from cave zones (entrance-CE, semidark-SD, dark-D) of Mediterranean subareas. For abbreviations of Mediterranean areas see Methods.

Considering the distribution of sponges along the light gradient inside caves, the highest total species richness was recorded in the semi-dark zone, followed by the dark and the cave entrance zone (Table S3). Such information existed for 85% of the sponge species found in caves. When the sponge assemblages inhabiting the three cave zones were compared for their similarity (Fig. 8), they were grouped according to the area and not the cave zone. Exceptions were the cave entrance zone of the French coastal caves and the dark zone of the North Aegean caves, both of which were separated from the other two zones of the corresponding areas (dissimilarity greater than 70%). In all areas, the two inner cave zones (semi-dark and dark) showed more similarity between each other (similarities reaching 75%) than with the cave entrance zone.

#### Biogeographical considerations

**Figure 9:**
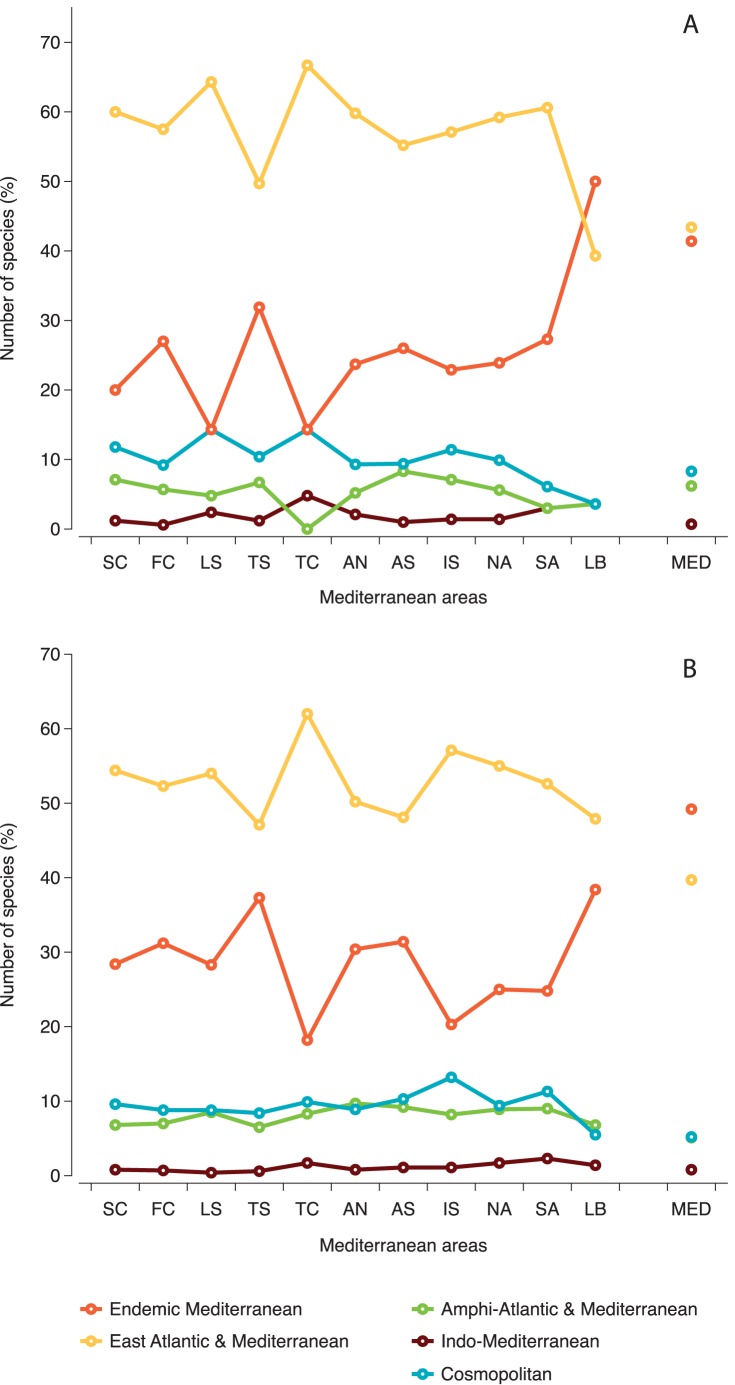
Zoogeographic characterization of sponge species in Mediterranean subareas and throughout the Mediterranean. (A) cave fauna, (B) overall Mediterranean fauna. For abbreviations of Mediterranean areas see Methods.

Out of the 290 species found in caves, 43.4% had an Atlanto-Mediterranean distribution and 41.4% were Mediterranean endemics (Table S3, Fig. 9A). Among the Atlanto-Mediterranean species, only a small number had an amphi-Atlantic distribution, while small percentages of Cosmopolitan and Indo-Mediterranean species were calculated. Examining the Mediterranean sponge fauna as a whole (Table S3, Figure 9B), endemics were the prevailing group (around 50%), while a slight decrease in the percent contribution of Atlanto-Mediterranean and Cosmopolitan species was observed. In both cases, the percentage of species with an Indo-Mediterranean distribution is negligible. A general northwest to southeast decline in the percentages of species with Atlanto-Mediterranean and cosmopolitan distribution was observed with a corresponding increase of the endemic component. The latter was highest in the Levantine caves, where the species with East Atlantic-Mediterranean distribution were very poorly represented in comparison to the other areas. Moreover, the caves of the Levantine and the Tunisian coast hosted the greatest percentage of Indo-Mediterranean species. This zoogeographic pattern of cave species was similar to that observed for the overall Mediterranean sponge fauna (Fig. 9B).

**Figure 10:**
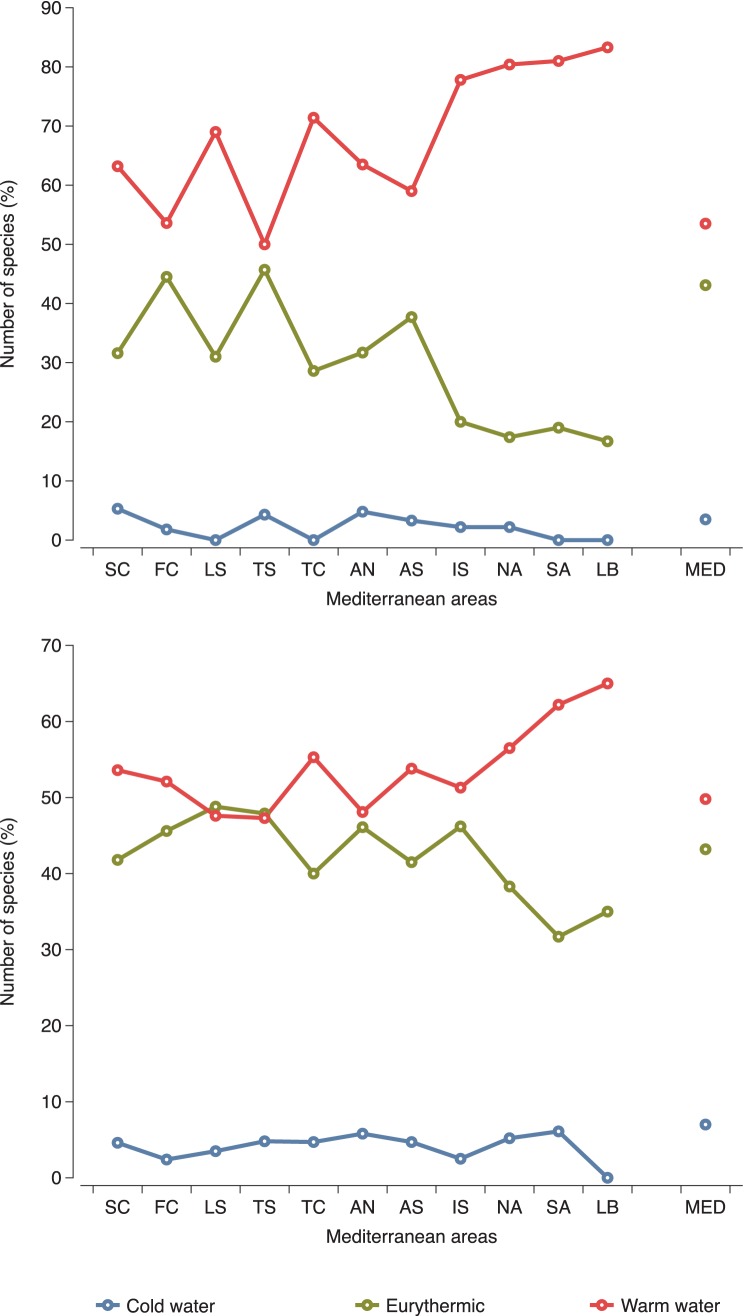
Bioclimatic categories of sponge species in Mediterranean subareas and throughout the Mediterranean. (A) cave fauna, (B) overall Mediterranean fauna. For abbreviations of Mediterranean areas see Methods.

A further zoogeographical analysis of the Atlanto-Mediterranean sponge species found in caves (Fig. 10A) showed that most of them (53.5%) had a distribution in warm regions, only few (3.5%) were cool region species, the remaining being widely spread all along the Eastern Atlantic coast (eurythermic species). The same analysis performed on the overall Mediterranean sponge fauna (Fig. 10B) revealed a slightly lower contribution of warm water species (49.8%) versus a higher contribution of cool water species (7%). The distribution of cool and warm water species for both cave sponges and the total sponge fauna in the Mediterranean areas showed a complementary pattern, with the cool water species percentage generally decreasing and the warm water species one increasing towards the southeastern areas.

## Discussion

Marine shallow water caves have been studied in the Mediterranean Sea (mostly in its northwestern basin) more intensively than anywhere else in the world ocean. This can be partly attributed to the development of diving techniques in this area and the early underwater marine surveys along the coasts of mostly France and Italy. A strong stimulus for the exploration of marine caves is that they are an integral part of the coastline in most areas of the Mediterranean, because of the extended distribution of limestone and karst systems [2].The use of Porifera for evaluating the significance of marine caves for the Mediterranean ecosystem has given interesting results highlighting both the representativeness and the uniqueness of cave sponge fauna.

### Species and Higher-taxon Richness

In the sponge assemblages found in Mediterranean caves, all poriferan classes and orders, 88 and 70% of the Demospongiae and Homoscleromorpha families and genera, respectively, and around half of their species are represented. Caves appear as an extremely favorable environment for certain groups, hosting, for instance, more than 70% of the Mediterranean diversity of Homoscleromorpha, Dictyoceratida, and Lithistida, as well as 20% of the global diversity of the former. The accumulation curves for sponge species recorded from surveyed Mediterranean marine caves (including cave-exclusive species) showed that no asymptote has been yet reached, indicating that there is still much to be discovered. Additionally, a plethora of specimens posing difficulties to identification, which are likely indicators of unknown hidden diversity, has been recorded at the genus level by various authors [17], [70] and the present study, paving the way for intensive research by sponge taxonomists.

### Regional Patterns of Diversity

Sponge species richness and affinities among cave assemblages of the different areas seem to follow a pattern quite similar to that of the overall Mediterranean sponge assemblages. Thus, most of the north Mediterranean areas had high cave species richness and were grouped together since they exhibit the most extensive carbonate rocky coasts (see Lewin & Woodward [2] for a detailed cartographic depiction). A divergence was observed in the case of the Ligurian Sea, which was separated from the above group, since it presents low cave species richness, most probably because of its limited rocky and karstic formations [71] and small number of relevant studies, when compared to other neighboring west Mediterranean areas. However, species richness of sponges found in caves from different Mediterranean areas was generally positively correlated with research effort, indicating that cave sponge fauna of some areas, rich in coastal rocky substrata, such as the North Aegean, South Aegean, and the Ionian Sea, might have been underestimated in the past. This is supported by our results from four North Aegean caves, which brought to light a notably species-rich sponge fauna. The persistent grouping of the North Aegean with the Spanish coasts according to the composition of their cave assemblages could be rather attributed to the fact that in both areas mainly insular caves were examined; the Balearic Islands, mostly explored for their caves, share similar environmental characteristics with the Aegean Archipelago: being far from the continental self they exhibit an oligotrophic character (higher temperatures and impoverished nutrients, plankton, and organic matter) and a proliferation of subtropical species, even a greater development of horny sponges [72]. Similar environmental characteristics might explain the low species richness recorded in Tunisian and Maltese cave assemblages, all of which were located in islands surrounded by the warm, oligotrophic, south Mediterranean waters. The Levantine and the South Aegean cave sponge communities are separated from all other cave communities; besides their low number of species recorded due to the limited research effort, this might be attributed to warm water affinities of their sponge fauna.

In addition to the numbers of studies and caves surveyed in each area, regional patterns of diversity may be affected by other features related to the breadth and depth of cave research, such as the aim and focus of the study, expertise of the research team, cave habitat coverage, duration of underwater research, etc. The above parameters are not homogenous at least for sponge research in the Mediterranean subareas and are difficult to quantify.

The values of taxonomic distinctness (AvTD) followed a regional pattern contrasting to that of species richness, being higher in the south-eastern areas and lower in the species-rich French and Tyrrhenian coasts. The high AvTD values calculated for the overall sponge fauna of the southeastern Mediterranean areas have been attributed to the relative climatic stability they experienced over the past 5 million years, and the small sea surface temperature anomalies that occurred there in comparison to the northwest Mediterranean areas [45]. Moreover, the normal values of VarTD calculated for the cave assemblages of this area may reflect the focused study of certain cave assemblages, which disclosed the expected uneven representation of higher taxa; interestingly, 45% of the families and 54% of the genera found in the Νorth Aegean were not represented in marine caves, while several others, such as Chalinidae, Irciniidae, and *Oscarella*, were overrepresented. On the other hand, the low values of VarTD for the overall Aegean sponge assemblages are probably due to their fragmentary, mostly faunistic study in the other sublittoral habitats. This is supported by the opposite trend (normal VarTD values for the overall sponge assemblages and low for the cave assemblages) calculated for the French and Tyrrhenian coasts, where extensive research has been carried out on the structure of hard-substrate sublittoral communities [73], [74].

Taxonomic distinctness was generally higher for cave assemblages than for the overall sponge assemblages in almost all the Mediterranean areas, mostly reflecting the lower ratio of species to genera in the former (S/G = 2.2 versus 3.2). For instance, several speciose genera, such as *Haliclona, Clathria, Hymedesmia,* and *Axinella* are considerably underrepresented in caves, with only half or one third of their Mediterranean species. It seems that marine caves favor the survival of particular species from diverse genera and families, hence reinforcing high taxonomic diversity. The dispersal of sessile invertebrate larvae from more or less distant parental habitats is determined by both environmental factors (e.g. hydrodynamic forces) and larval behaviour (e.g. swimming efficiency and phototaxis) [75], but subsequently, various environmental factors (e.g. light, food availability, and spatial competition) critically affect post-settlement mortality of propagules and larvae in semi-dark and dark cave environments, as shown by experimental research [16], [76]. The cave environment seems to be more favorable for a taxonomically broader sponge assemblage than the surrounding sublittoral rocky cliffs exposed to various disturbances, both natural [77], [78] and anthropogenic [79]; this is also indicated by other estimates of sponge diversity, which was found to decrease under turbulent and fast flow conditions [80]. Such contrasting patterns of biodiversity (low species richness versus high taxonomic distinctness) have been also observed in “stressful” habitats, such as hydrothermal vents, in relation to the surrounding environment [81].

Faunal similarities were much lower between areas when cave sponge assemblages were compared, than when their overall sponge fauna was taken into account, most probably reflecting the fragmentation of the cave habitat in the Mediterranean Sea. Fragmented habitats usually host species with strict habitat requirements and philopatric larval dispersal [82], though several of them have developed specific ways of dispersal to colonize new areas [83]. The strong influence of natural extreme fragmentation of the marine cave environment on population structure (high levels of genetic diversity and genetic structuring) was shown for other cave-dwelling invertebrates [84].

### Endemism and Zoogeographic Affinities

Caves altogether harbor more than 40% of the Mediterranean endemic Demospongiae and Homoscleromorpha species, this percentage becoming much higher for the latter and the demosponge order Lithistida. The lower endemic component for each subarea separately can be attributed to the narrow distribution of most endemic species (more than 70% of the endemics have been reported from 1 or 2 areas), in contrast to the wider distribution of Atlanto-Mediterranean species throughout the basin. The distribution of the endemic sponges in the caves of each Mediterranean area seems to reflect the research effort invested. Thus, Mediterranean endemics constitute more than 50% of the species recorded from the Levantine, by far greater than the endemic component of any other area, indicating the focused research carried out recently in this area by sponge specialists [28], [85]. Similarly, the low endemic percentage reported from the Ligurian Sea is due to the limited corresponding cave research, as mentioned above, while several Mediterranean neoendemics related to well-known Atlantic species have been recently added to the cave fauna of the French and Adriatic coasts [60]. These observations confirm previous speculations [52] that intensive research on sponges of underexplored areas (Eastern Mediterranean) and habitats (e.g. deep waters, caves) could change our knowledge concerning Mediterranean endemics.

The slightly higher total Atlanto-Mediterranean and Cosmopolitan components of cave sponge fauna in relation to the overall Mediterranean fauna might be associated with the fact that euryoecious species are more easily established in such peculiar environments, even though they may later develop populations with narrow ecological amplitude [86]. However, cosmopolitanism has been repeatedly questioned for sessile animals, such as sponges and it is highly expected that future taxonomic research will increase the endemic percentages among both cave and overall Mediterranean sponge fauna [87].

Zoogeographical analysis preformed in the present study showed that cave sponge assemblages of the Mediterranean seem to follow the pattern of the overall Mediterranean sponge fauna concerning the temperature range of species distribution. As for the Mediterranean Sea in general, cool water species are very restricted in Mediterranean caves, which appear to favor warm water assemblages. This results from the consideration of both the Mediterranean endemics and the greatest part of the species with an Eastern Atlantic-Mediterranean distribution; these two sponge faunal elements of the Mediterranean together constitute around 70% of Demospongiae and Homoscleromorpha diversity found in marine caves. The warm-water affinity of the cave fauna is further highlighted by the strong participation in this habitat of Dictyoceratida and Homoscleromorpha, which are represented in the Mediterranean by genera and species with a warm water and tropical distribution [88]. This situation is expected to be intensified through the ongoing tropical re-colonization of the Mediterranean due to the warming of its basin and the communication with the Indo-Pacific realm through the Suez Canal [89].

### Zonation of Sponge Assemblages in Caves

Marine cave assemblages are an integral part of the Mediterranean sciaphilous hard substrate communities. They are morphologically complex ecosystems with strong environmental gradients, therefore supporting a variety of different assemblages ranging from more or less sciaphilic algal dominated communities near the more illuminated entrance, to totally dark internal zones and even ‘azoic’ bare rock [12]. Along the cave gradient, three distinct biocoenoses have been defined and described by Pérès and Picard [90], i.e. the coralligenous (C), semi-dark (GSO), and totally dark caves (GO). The first one is a widely distributed Mediterranean biocoenosis, dominated by sciaphilic coralline algal bioconcretions, which frequently form coralligenous rims at the entrance part of marine caves [91].

The distinction in the present study of three major cave zones, namely the illuminated cave entrance (CE), semi-dark (SD), and dark (D) zone, was based on light intensity data recorded in the studied literature. In any case, it should be taken into consideration that the definition of ‘darkness’ is subjective and may vary with the authors. Thus, dark and totally dark zones have been distinguished in some cases [48], while light intensity along the different cave zones may exhibit fluctuations determined by season or micro-topography, resulting in spatial patchiness of the sessile cave biota [92]. Moreover, there are several other environmental features, crucial for the distinction and zonation of cave assemblages, such as the trophic conditions and hydrodynamism [92], which are scarcely described in the literature. Examined for their affinities, the three cave zones of each Mediterranean area were grouped together in most cases, indicating that regional differentiation is more influential than local ecological zonation. Furthermore, the two inner cave zones seem to provide a more or less similar environment for sponges in almost all areas, while the entrance zone is discretely different in all cases, probably because of its coralligenous nature. The pronounced differentiation of the cave entrance zone of the French coasts could be explained by the fact that the relevant data are derived from practically one cave entrance (Cave Niolon), characterized by high algal and gorgonian coverage and few sponge species [14].

In contrast to the general zonation pattern, semi-dark assemblage was more similar to the cave entrance coralligenous one in the North Aegean. The composition of the coralligenous communities from the eastern Mediterranean basin has been earlier discussed by Pérès and Picard [93], who observed that they were very rich in sponges and almost devoid of alcyonarians and gorgonians. This is in accordance with our observations in the North Aegean caves, which were ranked among the richest in sponges Mediterranean caves. The marked deviation of the dark zone, which presented a comparably low species richness, could be attributed to regional peculiarities associated with the relative oligotrophy of the Eastern Basin combined with the water circulation patterns; the caves studied are located in the central and eastern part of the North Aegean, thus subjected to influence of the saline and warm current, flowing from the Levantine northwards [94], [95].

### Unique Characteristics of Cave Sponge Diversity

The significance of the marine cave environment for the Mediterranean sponge biodiversity is further manifested through several unique characteristic features, highlighted by our results. One of these is the substantial number of cave-exclusive species, which becomes very high for certain groups, reaching 46 and 32% for Lithistida and Homoscleromorpha, implying that caves constitute a critical environment for their assemblages.

The recent advances in sponge taxonomy, using cytological, chemical and molecular characters are increasing our understanding of sponge diversity in marine caves. Species previously unknown such as *Thymosiopsis cuticulatus* and *Myceliospongia araneosa* were described [96] and divergence at the species level was revealed for closely related morphotypes. Examples of the latter case exist in Homoscleromopha and specifically in the genera *Plakina* and *Oscarella* to which several new species were assigned [59], [97], [98] but see Dailianis *et al.* [99].

The very limited distribution of most cave-exclusive species, with 68% of them found in only one and 26.5% in less than 5 caves, is one more indication of the high individuality and fragmentation of this habitat, furthermore reinforcing the opinion that marine caves possibly function as islands supporting isolated populations [92], [97]. However, the recent discovery of the carnivorous demosponge *Asbestopluma hypogea*, previously found only in dark cold water zones of caves [100], in the deep western Mediterranean basin (100–700 m) by Aguilar *et al.* [101], might imply that certain cave-exclusive species have evolved in deeper areas and colonized shallow cold water caves drifted by strong upwelling currents, as proposed for other cave-dwelling sponges [25] and bryozoans [102] from the same region. The presence of bathyal and eurybathic species in marine caves implies affinities of the cavernicolous sponge fauna with that of the lower continental shelf and slope (offshore rocky bottoms, deep-sea corals, etc.), where several of these species often form dense populations. Affinities of this type have been previously reported [24], [103].

Moreover, the exploration of marine and anchihaline caves with a descending or vertical profile in the French and Croatian coasts revealed the presence of the stenothermic deep-sea hexactinellid *Oopsacas minuta* along with other bathyal species [25], [104], [105], leading to the hypothesis that aphotic caves, whose topography favors trapping of cold water masses, thus providing stable low temperatures year-round (13–14.5°C), may function as ‘mesocosms’ of deep-sea conditions in the shallow zone [26].

Besides their unique faunal composition and bathyal affinitties, the Mediterranean caves exhibit another particular characteristic: they serve as potential refuges for ancient faunal elements [92]. A characteristic example is the calcareous sponge *Petrobiona massiliana*, whose finding resulted in the description of a new monotypic genus related to the Mesozoic fossil order Pharetronida [27]. The presence of *P. massiliana* in distant karstic areas matches a biogeographic model of disjunct distribution with metapopulations isolated in cryptic habitats of the western and central Mediterranean Sea, while fossils of this species have been found in Apulia (Italy) and the Island of Crete [106]. Moreover, during the last decade, several new species of the order Lithistida have been reported from shallow water caves, including possible i) relictual species or palaeoendemics [29], [107], and ii) remnants of the subtropical/tropical assemblages, which thrived in the Mediterranean during warm periods of its history and survived only in the warmer southeastern basin during the subsequent cooling episodes [28].

### Conservation Issues and Concluding Remarks

As an integral component of karstic coasts, most marine caves are located in the northern Mediterranean, and their protection becomes crucial since this area suffers the highest anthropogenic impact [108]. Semi-submerged and submerged marine caves are protected by the EC (Habitat Directive 92/43/EEC, Habitat type 8330). A recent evaluation by Abdulla *et al.* [109] has shown that around 30% of the Mediterranean MPAs include marine caves. However, the accurate number of marine caves within designated MPAs remains unknown while approximately 30% of those surveyed for their sponge fauna (e.g. Túnel Llarg in Medes Islands and Bagaud cave in Port-Cros National Park) are estimated to be embodied in MPAs. The need for their protection becomes more compelling under the pressure of ongoing climate change, strongly affecting the Mediterranean biodiversity [110]. During the last decades, disease outbreaks and mass mortality events, generated or enforced by temperature anomalies, have severely affected sessile benthic communities, mainly sponge and anthozoan populations in the north-western [111], [112] and eastern Mediterranean [113]. The environmental features of the cave habitat can provide to its assemblages a natural protection from possible human induced disturbances by mitigating temperature changes occurring outside, as well as exposure to pollutants and potential pathogens, as Garrabou *et al*. [114] showed for red coral populations in Provence. Furthermore, the clear biogeographic patterns exhibited by the Mediterranean marine cave assemblages from different regions, as shown in the present study, points out the need for protecting marine caves in each area of this semi-enclosed marine basin, in order to conserve representative sections of every sub-region.

Mediterranean marine caves constitute significant reservoirs of sponge species richness, phylogenetic diversity, and endemism, highly representative of the overall poriferan Mediterranean fauna. They serve as reservoirs of genetic diversity for rare species - many of which have not yet been described -, species that are cave-exclusive or have very limited range of distribution, living fossils, as well as species of economic interest. Many invertebrate species living in marine caves, mainly sponges, ascidians, and anthozoans, constitute proven or promising sources of bioactive compounds [115]. The four Mediterranean species of commercial bath sponges have been reported from caves, more or less frequently, with *Spongia officinalis* and *Hippospongia communis* being the most widely distributed. Cave populations of bath sponges might serve as a source of stock regeneration, as Dailianis *et al*. [99] suggested for their deeper populations, since they are less susceptible to both temperature-induced stress and harvesting pressure. Moreover, marine caves might be used for sponge culture, due to the limited sediment deposition and illumination [116]. All the above, emphasize the ecological and socio-economic value of marine caves, which constitute fundamental criteria for conservation policies [117], [118].

Our study showed that marine cave communities are not equally surveyed throughout the Mediterranean basin and several areas, especially in the southern and eastern coasts, are almost totally unexplored (Alboran Sea, Algerian, Libyan, and Egyptian coasts) or poorly investigated for their sponge fauna (Tunisian Coast, South Aegean, Levantine Basin, Ligurian Sea). Although mostly dominated by accretion coasts, some parts of these areas (coasts of southern Turkey, Libya, and Lebanon) exhibit locally extensive carbonate rock outcrops [2] with marine cave formations, where recent research has given interesting findings [28], [85]. We also came across gaps in the knowledge of spatial and ecological patterns of cave sponge assemblages even in relatively well studied areas (e.g. the Ionian Sea). Since most research has been carried out in shallow water caves further exploration should also focus in deeper caves, which might reveal interesting relationships with the bathyal fauna. Finally, similar studies are needed for other cave-dwelling organisms and an overall evaluation of Mediterranean marine cave biota is already under way.

## Supporting Information

Table S1 **Sponge species found in Aegean Sea caves during the present work.**
(PDF)

Table S2 **Poriferan fauna of Mediterrnean marine caves.**
(PDF)

Table S3 **Characteristic metrics of cave and overall Demospongiae and Homoscleromorpha fauna for each Mediterranean area.**
(PDF)

Table S4 **Mediterranean caves presenting the highest Poriferan species richness.**
(PDF)

Text S1 **Catalogue of literature on Mediterranean cave sponges.**
(PDF)
