# Supplementary material for: Marine Caves of the Mediterranean Sea: A Sponge Biodiversity Reservoir within a Biodiversity Hotspot
Source: PLoS One. 2012 Jul 11;7(7):e39873. doi: 10.1371/journal.pone.0039873 (PMC3394755; doi:10.1371/journal.pone.0039873)
Supplement: Table S1 — Sponge species found in Aegean Sea caves during the present work. (PDF) [file pone.0039873.s001.pdf]

Table S1. Sponge species found in Aegean caves during the present work.

SPECIES	CAVE ZONES	CAVES EXPLORED & PREVIOUS REFERENCES
Aaptos aaptos (Schmidt, 1864)	CE, SD	2, 4
Acanthella acuta Schmidt, 1862	CE, SD	1, 2, 3, 4, 5, 6, Voultsiadou (2005b)
Agelas oroides (Schmidt, 1864)	CE, SD, D	1, 2, 3, 4, 5, 6, 7, 8, 9, Voultsiadou (2005b)
Aplysilla rosea (Barrois, 1876)	CE, SD, D	1, 2, 5, 8
Aplysina aerophoba Nardo, 1843	CE, SD, D	4
Axinella cannabina (Esper, 1794)	SD	3
Axinella damicornis (Esper, 1794)	CE, SD	1, 3, 4, 5, 6, 8 Voultsiadou (2005b)
Axinella sp.	SD	3, 4
Axinella verrucosa (Esper, 1794)	SD	1, 3, 8, Voultsiadou (1986)
Bubaris sp.	D	3
Cacospongia mollior Schmidt, 1862	SD	2
Chondrosia reniformis Nardo, 1847	CE, SD, D	1, 2, 3, 4, 5, 6, 7, 9
Cliona celata Grant, 1826	CE	3, Voultsiadou (1986)
Cliona schmidti (Ridley, 1881)	CE, SD	1, 3, 4, 5
Coscinoderma sporadense Voultsiadou-Koukoura *et al*., 1991	SD	3, 4, Voultsiadou-Koukoura *et al*. (1991)
Crambe crambe (Schmidt, 1862)	CE	2, 6, Voultsiadou (1986)
Dendrilla sp.	SD, D	3, 4
Dendroxea lenis (Topsent, 1892)	SD	4
Dictyonella incisa (Schmidt, 1880)	SD, D	1, 2, 3, 4
Dictyonella obtusa (Schmidt, 1862)	D	4
Diplastrella bistellata (Schmidt, 1862)	D	3, Voultsiadou (2005b)
Dysidea avara (Schmidt, 1862)	CE, SD	3
Dysidea fragilis (Montagu, 1818	SD	3
Erylus euastrum (Schmidt, 1868)	CE, SD, D	3, 4, Voultsiadou (2005b)
Eurypon sp.	D	4
Eurypon clavatum (Bowerbank, 1866)	D	3
Fasciospongia cavernosa (Schmidt, 1862)	CE, SD	1, 5, 8, Voultsiadou-Koukoura & Koukouras (1993)
Geodia cydonium (Jameson, 1811)	SD	2, Pulitzer-Finali (1983)
Halichondria sp.1	SD	4
Halichondria sp.2	CE	2
Halichondrida sp.	SD, D	3, 4
**Haliclona (Gellius) microsigma* (Babic, 1922)	D	3
Haliclona (Halichoclona) fulva (Topsent, 1893)	D	3, 8
**Haliclona (Halichoclona) perlucida* (Griessinger, 1971)	SD	3
Haliclona (Soestella) mucosa (Griessinger, 1971)	CE, SD, D	1, 3, 4, 7
Haliclona sp.1	SD, D	1
Haliclona sp.2	SD	3
Haliclona sp.3	D	3
Haliclona sp.4	SD, D	1, 3
Haliclona sp.5	SD, D	1
***Hexadella pruvoti* Topsent, 1896	CE, SD, D	3, 4, 6
**Hexadella racovitzai* Topsent, 1896	CE, SD, D	3, 4
Hyrtios collectrix (Schulze, 1880)	CE	3
Ircinia oros (Schmidt, 1864)	CE, SD	1, 2, 3, 5, 6, 7, 9
Ircinia paucifilamentosa Vacelet, 1961	CE, SD, D	1, 3, 4, Vacelet (1961)
Ircinia variabilis (Schmidt, 1862)	CE, SD	1, 2, 3, 6, 8, Pulitzer-Finali & Pronzato (1981)
Jaspis johnstoni (Schmidt, 1862)	SD, D	1, 2, 3, 4, 5, 6, Voultsiadou 2005b
Jaspis sp.	D	4
Lithistida sp.	SD, D	1
Myrmekioderma spelaeum (Pulitzer-Finali, 1983)	SD, D	1, 6, 7, Voultsiadou & Vafidis (2004)
Oceanapia sp.	SD	3
**Oscarella balibaloi* Pérez *et al*., 2011	CE, SD, D	3, 4
**Oscarella microlobata* Muricy *et al*., 1996	D	3
***Oscarella tuberculata* (Schmidt, 1868)	SD	4
Penares helleri (Schmidt, 1864)	SD	3, Voultsiadou (2005b)
Petrosia (Petrosia) ficiformis (Poiret, 1789)	CE, SD, D	1, 2, 3, 6, 8, Jones *et al.* (1968)

Petrosia (Strongylophora) vansoesti Boury-Esnault *et al.*, 1994	SD, D	1, Voultsiadou & Vafidis (2004)
Phorbas tenacior (Topsent, 1925)	CE, SD	1, 2, 3, 4, 5, 6, 7, 8, 9, Pulitzer-Finali (1983)
Phorbas topsenti Vacelet & Perez, 2008	CE, SD	5
**Plakina bowerbankii* (Sarà, 1960)	SD, D	3, 4
Plakina monolopha Schulze, 1880	SD, D	4, Voultsiadou 2005b
Plakina sp.	SD, D	3, 4
Plakina trilopha Schulze, 1880	SD, D	4
Plakinidae sp.	D	3
Pleraplysilla spinifera (Schulze, 1879)	CE, SD	3, 4, 9
**Protosuberites rugosus* (Topsent, 1893)	D	3
**Pseudocorticium jarrei* Boury-Esnault *et al.*, 1995	SD, D	3, 4
Sarcotragus foetidus Schmidt, 1862	CE, SD, D	1, 3, 4, Voultsiadou (2005a)
Sarcotragus spinosulus Schmidt, 1862	SD	2
Spirastrella cunctatrix Schmidt, 1868	CE, SD, D	1, 2, 3, 4, 5, 6, 7, 8, 9, Jones *et al.* (1968)
Spongia (Spongia) nitens (Schmidt, 1862)	SD	3, 4
Spongia officinalis Linnaeus, 1759	CE, SD	1, 2, 3
Spongia (Spongia) virgultosa (Schmidt, 1868)	SD, D	4
Spongionella pulchella (Sowerby, 1804)	SD	3
Spongosorites sp.	CE, SD	3
Terpios gelatinosa (Bowerbank, 1866)	CE, SD, D	1, 2, 5, 8
***Thymosiopsis cuticulatus* Vacelet & Perez, 1998	SD, D	1, 3, 5, 6, 7
Timea geministellata Pulitzer-Finali, 1978	SD	2
Timea sp.	D	4
Timea unistellata (Topsent, 1892)	SD, D	4

* New records for the sponge fauna of the Aegean Sea.
** New records for the Eastern Mediterranean.
Caves explored: 1-Trypia Spilia, 2-Ftelio, 3-Fará, 4-Agios Vasilios, 5-Madhes, 6-Alykes, 7-Stavros, 8-Andros, 9-Vouliagmeni. Indicative referentes are given where available.
Cave zones: CE, cave entrance; SD, semi-dark zone; D, dark zone.
