# Supplementary material for: Marine Caves of the Mediterranean Sea: A Sponge Biodiversity Reservoir within a Biodiversity Hotspot
Source: PLoS One. 2012 Jul 11;7(7):e39873. doi: 10.1371/journal.pone.0039873 (PMC3394755; doi:10.1371/journal.pone.0039873)
Supplement: Table S2 — Poriferan fauna of Mediterrnean marine caves. (PDF) [file pone.0039873.s002.pdf]

Table S2. Poriferan fauna of Mediterranean marine caves.

SPECIES	MEDITERRANEAN SUBAREAS											NUMBER OF CAVES	CAVE ZONES
	SC	FC	LS	TS	TC	AN	AS	IS	NA	SA	LB		
Class **HOMOSCLEROMORPHA**													
Order **HOMOSCLEROPHORIDA**													
Family OSCARELLIDAE													
Oscarella balibaloi Pérez, Ivanisevic, Dubois, Pedel, Thomas, Tokina & Ereskovsky, 2011	+	+							+			4	CE, SD, D
Oscarella lobularis (Schmidt, 1862)	+	+	+	+	+	+	+	+	+			30	CE, SD, D
**Oscarella microlobata* Muricy, Boury-Esnault, Bézac & Vacelet, 1996		+							+		+	4	D
Oscarella tuberculata (Schmidt, 1868)	+	+							+			6	SD, D
**Oscarella viridis* Muricy, Boury-Esnault, Bézac & Vacelet, 1996		+										2	SD, D
**Pseudocorticium jarrei* Boury-Esnault, Muricy, Gallissian & Vacelet, 1995		+							+		+	5	SD, D
Family PLAKINIDAE													
Corticium candelabrum Schmidt, 1862	+	+		+		+	+	+				14	SD, D
Placinolopha moncharmonti (Sarà, 1960)				+								1	D
Plakina bowerbanki (Sarà, 1960)				+		+	+	+	+			9	SD, D
**Plakina crypta* Muricy, Boury-Esnault, Bézac & Vacelet, 1998		+										1	D
Plakina dilopha Schulze, 1880	+	+		+				+				4	SD
**Plakina endoumensis* Muricy, Boury-Esnault, Bézac & Vacelet, 1998		+										1	SD
**Plakina jani* Muricy, Boury-Esnault, Bézac & Vacelet, 1998		+										1	SD
Plakina monolopha Schulze, 1880		+		+					+			8	SD, D
Plakina reducta (Pulitzer-Finali, 1983)								+			+	3	
Plakina topsenti (Pouliquen, 1972)		+		+								3	D
Plakina trilopha Schulze, 1880	+	+		+			+	+	+			18	CE, SD, D
**Plakina weinbergi* Muricy, Boury-Esnault, Bézac & Vacelet, 1998										+	+	2	
Plakinastrella copiosa Schulze, 1880		+							+			4	SD, D
Plakortis simplex Schulze, 1880	+	+		+			+	+			+	19	CE, SD, D
Class **DEMOSPONGIAE**													
Order **SPIROPHORIDA**													
Family TETILLIDAE													
Cinachyrella levantinensis Vacelet, Bitar, Carteron, Zibrowius & Perez, 2007											+	2	
Craniella cranium (Müller, 1776)							+					1	
**Tetilla repens* (Sarà, 1958)				+								1	SD
Family SAMIDAE													
Samus anonymus Gray, 1867				+			+	+				5	CE
Order **ASTROPHORIDA**													
Family ANCORINIDAE													
Holoxea furtiva Topsent, 1892						+						1	D
Jaspis johnstoni (Schmidt, 1862)	+	+		+		+	+	+	+	+	+	25	CE, SD, D
Penares candidata (Schmidt, 1868)	+											1	
Penares helleri (Schmidt, 1864)	+	+	+	+		+	+	+	+		+	29	CE, SD, D
Stelletta grubii Schmidt, 1862			+	+			+					7	CE, SD, D
Stelletta lactea Carter, 1871		+		+								6	CE, SD, D
Stryphnus mucronatus (Schmidt, 1868)		+				+			+			4	SD, D
Family CALTHROPELLIDAE													
Calthropella (Calthropella) pathologica (Schmidt, 1868)	+	+										4	D

Family GEODIIDAE													
Caminus vulcani Schmidt, 1862				+								1	SD
Erylus discophorus (Schmidt, 1862)		+	+	+		+	+	+	+	+	+	32	CE, SD, D
Erylus euastrum (Schmidt, 1868)	+	+	+	+		+	+	+	+		+	29	CE, SD, D
Erylus mamillaris (Schmidt, 1862)				+								1	
Geodia conchilega Schmidt, 1862		+				+	+	+				8	SD, D
Geodia cydonium (Jameson, 1811)	+	+		+		+	+		+	+		20	SD, D
Isops intuta (Topsent, 1892)		+				+	+					7	SD, D
Family PACHASTRELLIDAE													
**Dercitus (Stoeba) dissimilis* (Sarà, 1959)				+								1	SD
Dercitus (Stoeba) plicatus (Schmidt, 1868)		+		+		+		+				8	CE, SD, D
Pachastrella monilifera Schmidt, 1868		+				+						5	SD, D
***Poecillastra compressa* (Bowerbank, 1866)				+								1	CE, SD
***Thenea muricata* (Bowerbank, 1858)				+								1	SD
Triptolemma simplex (Sarà, 1959)				+								1	SD
Family THOOSIDAE													
Alectona millari Carter, 1879		+		+			+	+				11	CE, SD, D
**Delectona madreporica* Bavestrello, Calcinai, Cerrano, Sarà, 1997			+					+				2	SD
Thoosa mollis Volz, 1939		+		+		+		+				5	CE, SD, D
Family THROMBIDAE													
Thrombus abyssi (Carter, 1873)		+										1	D
Order **HADROMERIDA**													
Family CLIONAIDAE													
Cliona celata Grant, 1826	+	+	+	+	+	+	+	+	+			20	CE, SD, D
Cliona janitrix Topsent, 1932				+								1	
Cliona rhodensis Rützler & Bromley, 1981						+	+	+				4	SD
Cliona schmidti (Ridley, 1881)	+			+		+	+	+	+	+	+	20	CE, SD, D
Cliona vermifera Hancock, 1867							+					1	D
Cliona viridis (Schmidt, 1862)	+	+	+	+		+	+	+		+		27	CE, SD, D
Cliothosa hancocki (Schmidt, 1862)				+		+						2	SD, D
Pione vastifica (Hancock, 1849)		+		+		+		+				6	SD, D
Spiroxya heteroclita Topsent, 1896				+								1	D
Spiroxya levispira (Topsent, 1898)		+		+								3	D
Volzia albicans (Volz, 1939)						+						1	SD, D
Family HEMIASTERELLIDAE													
Hemiasterella aristoteliana Voultsiadou-Koukoura & Van Soest, 1991									+			1	SD
Paratimea loricata (Sarà, 1958)				+								1	D
Paratimea pierantonii (Sarà, 1958)				+								1	SD
Family PLACOSPONGIIDAE													
Placospongia decorticans (Hanitsch, 1895)				+		+	+	+			+	14	CE, SD, D
Family POLYMASTIIDAE													
Polymastia harmelini Boury-Esnault & Bézac, 2007		+										1	CE
Polymastia penicillus (Montagu, 1818)				+								1	CE, SD
Pseudotrachya hystrix (Topsent, 1890)		+										1	D
Family SPIRASTRELLIDAE													
Diplastrella bistellata (Schmidt, 1862)	+	+	+	+		+	+	+	+			27	CE, SD, D
Diplastrella ornata Rützler & Sará, 1962				+		+	+	+		+	+	10	D
Spirastrella cunctatrix Schmidt, 1868	+	+	+	+	+	+	+	+	+	+	+	61	CE, SD, D
Family SUBERITIDAE													
Aaptos aaptos (Schmidt, 1864)	+	+	+	+		+	+	+	+			31	CE, SD, D
Prosuberites longispinus Topsent, 1893		+		+		+						6	SD, D
Protosuberites denhartogi van Soest & de Kluijver, 2003		+		+			+					6	SD, D
Protosuberites rugosus (Topsent, 1893)									+			1	D
Pseudosuberites sulphureus (Bean in Bowerbank, 1866)				+								1	D
***Rhizaxinella pyrifera* (Delle Chiaje, 1828)				+								1	SD

Suberites carnosus (Johnston, 1842)	+	+	+	+								10	CE, SD, D
Suberites domuncula (Olivi, 1792)						+						1	
Terpios gelatinosa (Bowerbank, 1866)	+	+	+	+		+	+	+	+	+		26	CE, SD, D
Family TETHYIDAE													
Tethya aurantium (Pallas, 1766)		+		+		+	+					17	CE, SD, D
Tethya citrina Sarà & Melone, 1965	+			+		+						3	D
Family TIMEIDAE													
Timea bifidostellata Pulitzer-Finali, 1983						+						1	
Timea crassa (Topsent, 1900)		+										1	SD
Timea fasciata Topsent, 1934		+		+			+	+				12	CE, SD, D
Timea geministellata Pulitzer-Finali, 1978						+			+			2	SD
Timea stellata (Bowerbank, 1866)				+			+					3	CE, SD
Timea stellifasciata Sarà & Siribelli, 1960							+					1	SD
Timea unistellata (Topsent, 1892)		+		+		+	+	+	+			13	CE, SD, D
Family TRACHYCLADIDAE													
Trachycladus minax (Topsent, 1888)		+		+			+					6	CE, SD, D
Order **CHONDROSIDA**													
Family CHONDRILLIDAE													
Chondrilla nucula Schmidt, 1862		+		+		+	+	+				7	CE, SD, D
Chondrosia reniformis Nardo, 1847	+	+	+	+	+	+	+	+	+	+	+	43	CE, SD, D
Thymosiopsis conglomerans Vacelet, Borchiellini, Perez, Bultel-Poncé, Brouard & Guyot, 2000		+				+						2	CE
Thymosiopsis cuticulatus Vacelet & Perez, 1998		+				+			+	+		9	SD, D
Family HALISARCIDAE													
Halisarca dujardini Johnston, 1842	+	+	+	+								5	CE, D
Order **LITHISTIDA**													
Family SCLERITODERMIDAE													
Aciculites mediterranea Manconi, Serusi & Pisera, 2006				+								1	D
Microscleroderma lamina Perez, Vacelet, Bitar & Zibrowius, 2004											+	1	D
Family CORALLISTIDAE													
Neophrissospongia endoumensis Pisera & Vacelet, 2011		+										1	SD
Neophrissospongia nana Manconi & Serusi 2008				+								1	D
Neophrissospongia nolitangere (Schmidt, 1870)		+										2	D
Neophrissospongia radjae Pisera & Vacelet, 2011						+						1	
Neoschrammeniella bowerbankii (Johnson, 1863)		+										3	D
Family THEONELLIDAE													
Discodermia polymorpha Pisera & Vacelet, 2011	+	+				+	+		+			11	SD, D
Family SIPHONIDIIDAE													
Gastrophanella phoeniciensis Perez, Vacelet, Bitar & Zibrowius, 2004											+	1	D
Family DESMANTHIDAE													
Petromica (Petromica) grimaldii Topsent, 1898				+								1	D
Order **POECILOSCLERIDA**													
Family ACARNIDAE													
Acarnus tortilis Topsent, 1892		+		+		+	+					6	CE, SD, D
Family MICROCIONIDAE													
Antho (Acarnia) coriacea (Bowerbank, 1874)				+								1	D
Antho (Antho) involvens (Schmidt, 1864)		+	+	+			+	+				7	CE, SD, D
Clathria (Clathria) compressa Schmidt, 1862		+										2	SD, D
Clathria (Clathria) coralloides (Olivi, 1792)		+										1	
Clathria (Clathria) toxistricta Topsent, 1925		+										1	
Clathria (Clathria) toxistyla (Sarà, 1959)							+					4	CE
Clathria (Clathria) toxivaria (Sarà, 1959)		+	+	+			+	+				11	CE, SD
Clathria (Microciona) ascendens (Cabioch, 1968)	+											1	SD
Clathria (Microciona) duplex Sarà, 1958				+								3	SD
Clathria (Microciona) gradalis Topsent, 1925				+								1	

Clathria (Microciona) poecilosclera (Sarà & Siribelli, 1960)				+								1	D
Clathria (Microciona) spinarcus (Carter & Hope, 1889)		+										1	
Clathria (Microciona) strepsitoxa (Hope, 1889)		+										1	SD, D
Clathria (Microciona) toximajor Topsent, 1925				+								1	SD
Clathria (Microciona) toxitenuis Topsent, 1925		+	+	+			+					7	CE, SD
Clathria (Thalysias) jolicoeuri (Topsent, 1892)		+										3	CE, SD
Family RASPAILIIDAE													
Eurypon cinctum Sarà, 1960		+										1	
Eurypon clavatum (Bowerbank, 1866)		+							+			3	SD, D
Eurypon lacazei (Topsent, 1891)	+	+										6	SD, D
Eurypon major Sarà & Siribelli, 1960				+		+						2	CE
Eurypon vesciculare Sarà & Siribelli, 1960							+					3	CE, SD
Raspaciona aculeata (Johnston, 1842)	+	+		+		+						9	CE, SD, D
**Raspaciona calva* Sarà, 1958				+								1	CE, SD
Raspaciona robusta Sarà, 1958				+								2	CE, SD, D
Raspailia (Raspailia) viminalis Schmidt, 1862	+											1	
Family RHABDEREMIIDAE													
Rhabderemia gallica van Soest & Hooper, 1993		+										1	D
Rhabderemia spinosa Topsent, 1896		+				+	+					4	D
Rhabderemia topsenti van Soest & Hooper, 1993		+										1	SD, D
Rhabderemia toxigera Topsent, 1892		+										1	D
Family CHONDROPSIDAE													
Batzella inops (Topsent, 1891)	+	+		+			+					6	CE, SD
Family COELOSPHAERIDAE													
Forcepia (Leptolabis) luciensis (Topsent, 1888)		+										1	SD, D
Lissodendoryx (Anomodoryx) cavernosa (Topsent, 1892)				+								1	
Lissodendoryx (Lissodendoryx) basispinosa Sarà, 1958				+								1	CE
Family CRAMBEIDAE													
Crambe crambe (Schmidt, 1862)	+	+	+	+	+	+	+	+	+	+	+	44	CE, SD, D
Crambe tailliezi Vacelet & Boury-Esnault, 1982		+										2	
Family CRELLIDAE													
Crella (Crella) elegans (Schmidt, 1862)		+		+								3	CE, D
Crella (Grayella) pulvinar (Schmidt, 1868)	+	+		+								9	CE, SD, D
Crella (Pytheas) sigmata Topsent, 1925		+										1	SD, D
**Crella (Yvesia) nodulosa* Sarà, 1959				+								1	SD
Crella (Yvesia) rosea (Topsent, 1892)		+										3	SD, D
Crella (Yvesia) topsenti (Babiç, 1922)				+								1	
Family HYMEDESMIIDAE													
Hamigera hamigera (Schmidt, 1862)		+			+	+					+	4	
Hemimycale columella (Bowerbank, 1874)	+	+	+	+	+	+	+	+				22	CE, SD, D
Hymedesmia (Hymedesmia) baculifera (Topsent, 1901)		+										1	
Hymedesmia (Hymedesmia) castanea Sarà, 1964			+									2	CE
Hymedesmia (Hymedesmia) pansa Bowerbank, 1882		+		+								3	SD, D
Hymedesmia (Hymedesmia) peachi Bowerbank, 1882	+	+		+			+	+				6	D
**Hymedesmia (Hymedesmia) rectirhaphis* (Pulitzer-Finali, 1983)				+								1	
Hymedesmia (Hymedesmia) versicolor (Topsent, 1893)	+	+	+	+		+	+					10	CE, SD, D
Hymedesmia (Hymedesmia) zetlandica Bowerbank, 1864				+								1	SD
Hymedesmia (Stylopus) coriacea (Fristedt, 1885)		+		+		+						5	SD, D
Hymedesmia (Stylopus) nigrescens (Topsent, 1925)				+								1	
Phorbas dives (Topsent, 1891)		+		+								2	SD
Phorbas fibulatus (Topsent, 1893)		+		+								2	
Phorbas fictitius (Bowerbank, 1866)	+	+	+	+		+	+	+				14	CE, SD, D
Phorbas plumosus (Montagu, 1818)				+								1	
Phorbas tailliezi Vacelet & Perez, 2008							+					1	
Phorbas tenacior (Topsent, 1925)	+	+	+	+	+	+	+	+	+	+		54	CE, SD, D
Phorbas topsenti Vacelet & Perez, 2008			+	+						+		4	CE, SD
Family MYXILLIDAE													

Myxilla (Myxilla) iotrochotina (Topsent, 1892)		+		+				+				6	CE, SD
Myxilla (Myxilla) macrosigma Boury-Esnault, 1971		+										2	SD
Myxilla (Myxilla) rosacea (Lieberkühn, 1859)		+		+		+	+					5	SD, D
Family TEDANIIDAE													
Tedania (Tedania) anhelans (Lieberkühn, 1859)		+					+	+				3	CE
Family CLADORHIZIDAE													
Asbestopluma hypogea Vacelet & Boury-Esnault, 1996		+				+						3	CE, SD, D
Family HAMACANTHIDAE													
Hamacantha (Hamacantha) papillata Vosmaer, 1885		+										1	CE
Family MYCALIDAE													
Mycale (Aegogropila) contarenii (Martens, 1824)		+										1	
Mycale (Aegogropila) rotalis (Bowerbank, 1874)		+		+								4	CE, SD
Mycale (Aegogropila) tunicata (Schmidt, 1862)			+									1	CE, SD
Mycale (Carmia) macilenta (Bowerbank, 1866)		+										1	SD
Mycale (Mycale) lingua (Bowerbank, 1866)							+					1	CE
Mycale (Mycale) massa (Schmidt, 1862)		+		+		+						2	CE, D
**Mycale (Paresperella) dentata* Sarà, 1958				+								1	SD
Family MERLIIDAE													
Merlia deficiens Vacelet, 1980		+										1	SD
Merlia lipoclavidisca Vacelet & Uriz, 1991							+					1	
Merlia normani Kirkpatrick, 1908	+	+		+		+	+	+	+	+		16	CE, SD, D
Order **HALICHONDRIDA**													
Family AXINELLIDAE													
Axinella cannabina (Esper, 1794)						+		+	+			5	SD
Axinella damicornis (Esper, 1794)	+	+	+	+	+	+	+	+	+	+		35	CE, SD, D
Axinella polypoides Schmidt, 1862		+			+				+		+	8	CE, SD
Axinella rugosa (Bowerbank, 1866)	+											1	
Axinella vaceleti Pansini, 1984		+		+								3	SD
Axinella verrucosa (Esper, 1794)	+	+	+	+		+	+	+	+	+		20	CE, SD, D
Phakellia robusta Bowerbank, 1866		+										2	SD
Family BUBARIDAE													
Bubaris vermiculata (Bowerbank, 1866)				+		+	+	+				5	SD, D
Monocrepidium vermiculatum Topsent, 1898	+	+		+								3	SD, D
Family DICTYONELLIDAE													
Acanthella acuta Schmidt, 1862	+	+		+		+	+	+	+	+	+	31	CE, SD, D
**Acanthella annulata* Sarà, 1958				+								1	SD
Dictyonella incisa (Schmidt, 1880)		+		+		+	+	+	+			15	SD, D
Dictyonella marsilii (Topsent, 1893)							+		+			2	CE, SD
Dictyonella obtusa (Schmidt, 1862)		+							+			2	D
Dictyonella pelligera (Schmidt, 1864)		+		+								2	CE, SD
**Scopalina azurea* Bibiloni, 1993	+											1	D
Scopalina lophyropoda Schmidt, 1862		+										1	SD
Tethyspira spinosa (Bowerbank, 1874)				+								1	CE, SD
Family HALICHONDRIIDAE													
Amorphinopsis pallescens (Topsent, 1892)	+											1	
Axinyssa papillosa (Sarà & Siribelli, 1960)		+										1	D
Ciocalypta penicillus Bowerbank, 1862		+		+								4	SD
Halichondria (Halichondria) contorta (Sarà, 1961)	+			+			+					9	SD, D
Halichondria (Halichondria) genitrix (Schmidt, 1870)	+	+		+		+	+					6	SD, D
Halichondria (Halichondria) panicea (Pallas, 1766)		+		+								3	CE, SD
Halichondria (Halichondria) semitubulosa Lieberkühn, 1859	+			+		+	+					8	CE, D
Hymeniacidon mixta (Sarà, 1958)				+								1	
Hymeniacidon perlevis (Montagu, 1818)	+	+		+				+				8	CE, SD
**Spongosorites cavernicola* Bibiloni, 1993	+											1	D
Spongosorites flavens Pulitzer-Finali, 1983	+			+		+	+					7	CE, SD, D
Spongosorites intricatus (Topsent, 1892)		+		+								2	CE, D
**Topsentia garciae* Bibiloni, 1993	+											3	SD, D

Topsentia lacazei (Schmidt, 1868)											+	1	D
Family HETEROXYIDAE													
**Didiscus pseudodidiscoides* (Corriero, Scalera-Liaci & Pronzato, 1996)							+					1	SD, D
Didiscus spinoxeatus Corriero, Scalera-Liaci & Pronzato, 1997				+								2	CE, SD, D
Didiscus stylifer Tsurnamal, 1969								+			+	3	
Halicnemia geniculata Sarà, 1958		+		+								2	SD, D
Halicnemia patera Bowerbank, 1864	+	+		+								3	SD
**Higginsia ciccaresei* Pansini & Pesce, 1998								+				1	
Myrmekioderma spelaeum (Pulitzer-Finali, 1983)	+			+		+	+	+	+	+	+	12	SD, D
Order **AGELASIDA**													
Family AGELASIDAE													
Agelas oroides (Schmidt, 1864)	+	+	+	+	+	+	+	+	+	+	+	64	CE, SD, D
Order **HAPLOSCLERIDA**													
Family CALLYSPONGIIDAE													
Callyspongia subcornea Griessinger, 1971		+										3	CE, SD
Siphonochalina coriacea Schmidt, 1868		+										1	
Family CHALINIDAE													
Chalinula limbata (Montagu, 1818)				+								1	
**Dendrectilla tremitensis* Pulitzer-Finali, 1983							+					1	
**Dendroxea adumbrata* Corriero, Scalera-Liaci & Pronzato, 1996		+		+		+						4	SD
Dendroxea lenis (Topsent, 1892)	+	+		+				+	+			13	CE, SD, D
Haliclona (Gellius) angulata (Bowerbank, 1866)		+				+						2	
Haliclona (Gellius) dubia (Babic, 1922)							+					1	CE
Haliclona (Gellius) fibulata (Schmidt, 1862)		+		+		+	+					7	CE, SD, D
Haliclona (Gellius) lacazei (Topsent, 1893)	+	+										5	SD, D
Haliclona (Gellius) laxa (Topsent, 1892)		+										2	SD, D
Haliclona (Gellius) microsigma (Babic, 1922)		+							+			2	D
Haliclona (Halichoclona) fulva (Topsent, 1893)	+	+		+		+	+	+	+	+		21	SD, D
Haliclona (Halichoclona) parietalis (Topsent, 1893)							+					2	
Haliclona (Halichoclona) perlucida (Griessinger, 1971)									+			1	SD
Haliclona (Haliclona) simulans (Johnston, 1842)		+		+								2	CE
Haliclona (Haliclona) varia (Sarà, 1958)			+	+			+					13	CE, SD, D
Haliclona (Reniera) aquaeductus (Schmidt, 1862)		+						+				2	CE, SD, D
Haliclona (Reniera) cinerea (Grant, 1826)			+	+		+	+					9	CE, SD, D
Haliclona (Reniera) citrina (Topsent, 1892)		+										1	
Haliclona (Reniera) cratera (Schmidt, 1862)	+	+		+		+	+	+		+		19	CE, SD, D
Haliclona (Reniera) mediterranea Griessinger, 1971		+							+			3	SD
Haliclona (Reniera) subtilis Griessinger, 1971	+	+										3	CE
Haliclona (Rhizoniera) sarai (Pulitzer-Finali, 1969)	+	+		+		+	+	+				30	CE, SD, D
Haliclona (Soestella) mamillata (Griessinger, 1971)		+				+						2	
Haliclona (Soestella) mucosa (Griessinger, 1971)	+	+	+	+		+	+		+	+		26	CE, SD, D
Haliclona (Soestella) valliculata (Griessinger, 1971)	+	+		+								5	SD, D
Haliclona aperta (Sarà, 1960)		+										4	SD, D
Family PHLOEODICTYIDAE													
Calyx nicaeensis (Risso, 1826)		+		+			+					4	
**Oceanapia constructa* (Rützler, 1965)						+						1	D
Oceanapia decipiens (Sarà, 1958)				+								1	SD
**Oceanapia minor* (Sarà, 1958)				+								1	SD
**Oceanapia vacua* (Sarà, 1961)		+					+					2	CE, D
Family PETROSIIDAE													
Petrosia (Petrosia) ficiformis (Poiret, 1789)	+	+	+	+	+	+	+	+	+	+	+	67	CE, SD, D
Petrosia (Strongylophora) vansoesti Boury-Esnault, Pansini & Uriz, 1994									+			2	SD, D
**Petrosia pulitzeri* Pansini, 1996				+						+		2	
Order **DICTYOCERATIDA**													

Family IRCINIIDAE													
Ircinia dendroides (Schmidt, 1862)	+	+		+		+	+	+	+	+		15	CE, SD, D
Ircinia oros (Schmidt, 1864)	+	+	+	+	+	+	+		+	+		23	CE, SD, D
Ircinia paucifilamentosa Vacelet, 1961									+	+		6	CE, SD, D
Ircinia variabilis (Schmidt, 1862)	+	+	+	+	+	+	+	+	+	+	+	65	CE, SD, D
Sarcotragus foetidus Schmidt, 1862	+	+		+	+	+	+		+			11	CE, SD, D
Sarcotragus pipetta (Schmidt, 1868)				+								2	SD
Sarcotragus spinosulus Schmidt, 1862	+	+	+	+		+	+	+	+			18	CE, SD, D
Family THORECTIDAE													
Cacospongia mollior Schmidt, 1862	+	+		+					+			12	CE, SD, D
Fasciospongia cavernosa (Schmidt, 1862)		+		+	+	+	+	+	+	+		14	CE, SD
Hyrtios collectrix (Schulze, 1880)									+			1	CE
Scalarispongia proficens (Pulitzer-Finali & Pronzato, 1980)							+					4	
Scalarispongia scalaris (Schmidt, 1862)	+	+	+	+		+	+	+				24	CE, SD, D
Family SPONGIIDAE													
Coscinoderma sporadense Voultsiadou-Koukoura, van Soest & Koukouras, 1991									+			4	SD
Hippospongia communis (Lamarck, 1814)	+	+		+		+						11	CE, SD, D
Spongia (Spongia) lamella (Schulze, 1879)		+										1	SD
Spongia (Spongia) nitens (Schmidt, 1862)		+				+		+	+			7	CE, SD, D
Spongia (Spongia) officinalis Linnaeus, 1759	+	+	+	+	+	+	+	+	+			26	CE, SD, D
Spongia (Spongia) virgultosa (Schmidt, 1868)	+	+	+	+		+	+	+	+			38	CE, SD, D
Spongia (Spongia) zimocca Schmidt, 1862					+							1	
Family DYSIDEIDAE													
Dysidea avara (Schmidt, 1862)	+	+		+		+	+	+	+			16	CE, SD, D
Dysidea fragilis (Montagu, 1818)	+	+	+	+	+	+	+		+			20	CE, SD, D
Dysidea incrustans (Schmidt, 1862)			+	+				+				4	CE, SD
Dysidea tupha (Martens, 1824)				+	+							3	
**Euryspongia raouchensis* Vacelet, Bitar, Carteron, Zibrowius & Perez, 2007											+	1	
Pleraplysilla spinifera (Schulze, 1879)	+	+		+		+		+	+	+		20	CE, SD, D
Order **DENDROCERATIDA**													
Family DARWINELLIDAE													
Aplysilla rosea (Barrois, 1876)	+	+	+	+	+	+	+		+	+		25	CE, SD, D
Chelonaplysilla noevus (Carter, 1876)	+	+										4	SD, D
Family DICTYODENDRILLIDAE													
Spongionella gracilis (Vosmaer, 1883)				+								1	
Spongionella pulchella (Sowerby, 1804)	+	+							+			5	CE, SD
Order **VERONGIDA**													
Family APLYSINIDAE													
Aplysina aerophoba Nardo, 1843	+					+			+			4	CE, SD, D
Aplysina cavernicola (Vacelet, 1959)	+	+		+		+						14	CE, SD, D
Family IANTHELLIDAE													
Hexadella pruvoti Topsent, 1896	+	+							+	+		6	CE, SD, D
Hexadella racovitzai Topsent, 1896	+	+				+		+	+			6	CE, SD, D
Order **DEMOSPONGIAE INCERTAE SEDIS**													
Myceliospongia araneosa Vacelet & Perez, 1998	+	+										3	SD, D
Class **CALCAREA**													
Order **CLATHRINIDA**													
Family CLATHRINIDAE													
Clathrina cerebrum (Haeckel, 1872)		+										3	SD, D
Clathrina clathrus (Schmidt, 1864)	+	+	+			+		+			+	16	CE, SD, D
Clathrina contorta (Bowerbank, 1866)		+		+		+	+					11	CE, SD, D
Clathrina primordialis (Haeckel, 1872)		+										1	SD, D
Clathrina reticulum (Schmidt, 1862)		+										2	D
Clathrina rubra Sarà, 1958		+	+	+				+				7	CE, SD, D

Guancha blanca Miklucho-Maclay, 1868		+		+								5	CE, SD, D
Family LEUCALTIDAE													
Ascandra falcata Haeckel, 1870		+		+		+	+					10	CE, SD, D
Family LEUCETTIDAE													
Leucetta solida (Schmidt, 1862)		+	+	+		+	+				+	18	CE, SD, D
Order **LEUCOSOLENIDA**													
Family LEUCOSOLENIIDAE													
Leucosolenia variabilis (Haeckel, 1870)	+		+	+								4	CE, SD, D
Family AMPHORISCIDAE													
Amphoriscus salfii Sarà, 1951				+								1	SD, D
Family GRANTIIDAE													
Leucandra aspera (Schmidt, 1862)			+	+			+					7	CE, SD, D
Leucandra crambessa Haeckel, 1872				+								2	CE, SD, D
Ute glabra Schmidt, 1864		+										1	
Family SYCETTIDAE													
Sycon ciliatum (Fabricius, 1780)				+								2	CE, SD, D
Sycon elegans (Bowerbank, 1845)	+	+	+	+			+					7	CE, SD, D
Sycon humboldti Risso, 1826											+	1	
Sycon raphanus Schmidt, 1862		+	+	+								6	CE, SD, D
Order **LITHONIDA**													
Family MINCHINELLIDAE													
Monoplectroninia hispida Pouliquen & Vacelet, 1970		+										1	D
**Plectroninia hindei mediterranea* Vacelet, 1967		+										1	D
Family PETROBIONIDAE													
Petrobiona massiliana Vacelet & Lévi, 1958		+	+	+	+			+				18	SD, D
Class **HEXACTINELLIDA**													
Order **LYSSACINOSIDA**													
Family LEUCOPSACIDAE													
Oopsacas minuta Topsent, 1927		+				+						6	D

For Mediterranean subareas abbreviations see Methods. For cave zones abbreviations see Table S1.
* Species found exclusively in marine caves up to date.
** The presence of this species in marine caves, recorded by Russ & Rützler (1959), has been disputed by Vacelet (1969).
