# Supplementary material for: Marine Caves of the Mediterranean Sea: A Sponge Biodiversity Reservoir within a Biodiversity Hotspot
Source: PLoS One. 2012 Jul 11;7(7):e39873. doi: 10.1371/journal.pone.0039873 (PMC3394755; doi:10.1371/journal.pone.0039873)
Supplement: Table S3 — Characteristic metrics of cave and overall Demospongiae and Homoscleromorpha fauna for each Mediterranean area. (PDF) [file pone.0039873.s003.pdf]

Table S3. Characteristic metrics of cave and overall Demospongiae and Homoscleromorpha fauna for each Mediterranean area.

		SC	FC	LS	TS	TC	AN	AS	IS	NA	SA	LB	TOTAL
**Cave sponge fauna**	**Total species richness**	85	174	42	163	21	97	96	70	71	33	28	290
	**Endemic**	17 (20)	47 (27)	6 (14.3)	52 (31.9)	3 (14.3)	23 (23.7)	25 (26)	16 (22.9)	17 (23.9)	9 (27.3)	14 (50)	120 (41.4)
	**East Atl-Med**	51 (60)	100 (57.5)	27 (64.3)	81 (49.7)	14 (66.7)	58 (59.8)	53 (55.2)	40 (57.1)	42 (59.2)	20 (60.6)	11 (39.3)	126 (43.4)
	**Amphi Atl-Med**	6 (7.1)	10 (5.7)	2 (4.8)	11 (6.7)	0 (0)	5 (5.2)	8 (8.3)	5 (7.1)	4 (5.6)	1 (3)	1 (3.6)	18 (6.2)
	**Indo-Med**	1 (1.2)	1 (0.6)	1 (2.4)	2 (1.2)	1 (4.8)	2 (2.1)	1 (1)	1 (1.4)	1 (1.4)	1 (3)	1 (3.6)	2 (0.7)
	**Cosmopolitan**	10 (11.8)	16 (9.2)	6 (14.3)	17 (10.4)	3 (14.3)	9 (9.3)	9 (9.4)	8 (11.4)	7 (9.9)	2 (6.1)	1 (3.6)	24 (8.3)
	**AvTD (Δ+)**	96.25	95.46	95.18	95.47	94.17	95.98	95.73	96.04	95.88	96.35	97.02	-
	**VarTD (Λ+)**	138.45	151.88	175.66	150.56	213.00	138.06	148.20	146.03	163.07	163.08	105.23	-
	**Number of studies**	14	37	9	33	4	10	9	13	8	5	8	128
	**Number of caves**	11	25	14	39	8	20	20	19	9	8	12	185
	**Cave Entrance zone richness**	33	28	30	59	7	23	39	3	28	-	-	116
	**Semi Dark zone richness**	43	98	14	101	1	39	40	14	63	1	-	187
	**Dark zone richness**	52	85	-	72	-	44	34	2	32	-	3	170
**Mediterranean sponge fauna**	**Total species richness**	250	285	272	308	121	257	185	182	180	133	73	610
	**Endemic**	71 (28.4)	89 (31.2)	77 (28.3)	115 (37.3)	22 (18.2)	78 (30.4)	58 (31.4)	37 (20.3)	45 (25)	33 (24.8)	28 (38.4)	300 (49.2)
	**East Atl-Med**	136 (54.4)	149 (52.3)	147 (54)	145 (47.1)	75 (62)	129 (50.2)	89 (48.1)	104 (57.1)	99 (55)	70 (52.6)	35 (47.9)	242 (39.7)
	**Amphi Atl-Med**	17 (6.8)	20 (7)	23 (8.5)	20 (6.5)	10 (8.3)	25 (9.7)	17 (9.2)	15 (8.2)	16 (8.9)	12 (9)	5 (6.8)	31 (5.1)
	**Indo-Med**	2 (0.8)	2 (0.7)	1 (0.4)	2 (0.6)	2 (1.7)	2 (0.8)	2 (1.1)	2 (1.1)	3 (1.7)	3 (2.3)	1 (1.4)	5 (0.8)
	**Cosmopolitan**	24 (9.6)	25 (8.8)	24 (8.8)	26 (8.4)	12 (9.9)	23 (8.9)	19 (10.3)	24 (13.2)	17 (9.4)	15 (11.3)	4 (5.5)	32 (5.2)
	**AvTD (Δ+)**	95.02	94.67	94.63	94.60	94.54	95.05	95.44	95.32	95.74	95.87	96.42	-
	**VarTD (Λ+)**	169.10	165.62	168.09	173.42	183.38	169.99	158.90	157.07	146.03	143.48	122.29	-

Percentages are given in parentheses.
For Mediterranean subareas abbreviations and zoogeographic characterizations see Methods.
