# Supplementary material for: Marine Caves of the Mediterranean Sea: A Sponge Biodiversity Reservoir within a Biodiversity Hotspot
Source: PLoS One. 2012 Jul 11;7(7):e39873. doi: 10.1371/journal.pone.0039873 (PMC3394755; doi:10.1371/journal.pone.0039873)
Supplement: Table S4 — Mediterranean caves presenting the highest Poriferan species richness. (PDF) [file pone.0039873.s004.pdf]

Table S4. Mediterranean caves presenting the highest Poriferan species richness.

Cave's name (Region)	Species richness*	References
Grotte du Figuier (FC)	79-86	Vacelet 1967; Pouliquen 1969; Griessinger 1971; Pouliquen 1972; Vacelet & Boury-Esnault 1982; Pansini 1984; Pisera & Vacelet 2011
Grotte d' Endoume (FC)	74-75	Pouliquen 1972; Muricy *et al*. 1996b; Muricy *et al*. 1998; Vishnyakov & Ereskovsky 2009; Pisera & Vacelet 2011
Grotta della Gaiola (TS)	59-63	Sarà 1959b; 1960; 1961b; 1962b
Grotta di Mitigliano (TS)	58-75	Pansini & Pronzato 1982; Pulitzer-Finali 1983; Balduzzi *et al*. 1985; 1989; Pansini 1996
Grotte des Trémies (FC)	57-61	Pouliquen 1969; 1972; Pisera & Vacelet 2011
Grotta dei Misteri (TS)	55-56	Sarà 1958; 1959a; 1959b ; 1960; 1961b; 1962b
Bue Marino (AS)	54-59	Sará 1961a; Corriero *et al*. 1996; Corriero et al 2000
Grotta del Mago (TS)	47-51	Pulitzer-Finali 1970; Cinelli *et al*. 1977; Pansini *et al*. 1977; Pulitzer-Finali & Pronzato 1977; Pulitzer-Finali 1978
Tuffo Tuffo (TS)	45-46	Russ & Rützler 1959; Rützler 1965a
Grotte de Niolon (FC)	44-50	Pérès & Picard 1949; Laborel & Vacelet 1958; 1959; Vacelet 1959; Laborel 1960; Vacelet 1964; True 1970; Griessinger 1971
Grotta Verde (TS)	42-44	Corriero *et al*. 1997
Fará (NA)	41-53	Current study
Catedral (SC)	41-44	Bibiloni & Gili 1982; Bibiloni *et al*. 1989; Bibiloni 1993
Bagaud (FC)	39-47	Muricy *et al*. 1996a; Harmelin *et al*. 2003
Grotta delle Viole (AS)	39-43	Sará 1961a ; Pulitzer-Finali & Pronzato 1981; Pulitzer-Finali 1983
Túnel Llarg (SC)	33-38	Gili *et al*. 1982; Bibiloni *et al*. 1984
Misidacis (SC)	31-44	Martí *et al*. 2004
Agios Vasileios (NA)	30-39	Current study
Cova Blava (SC)	30-39	Uriz *et al*. 1992 ; Martí *et al*. 2004 ; Turon *et al*. 2009
Stražica Cape (AN)	30-32	Novosel *et al*. 2002
Cala Tonda (AS)	29-32	Sará 1961a; 1962b; Pulitzer-Finali 1983
Porto Cesareo (IS)	28-29	Corriero *et al*. 2004
Youra cave (NA)	26	Voultsiadou 1986; Voultsiadou-Koukoura & Soest 1991; Voultsiadou-Koukoura *et al*. 1991; Voultsiadou-Koukoura & Koukouras 1993; Voultsiadou & Vafidis 2004; Voultsiadou 2005b
Trois Pépés (FC)	23	Vacelet *et al*. 1994; Corriero *et al*. 1996; Muricy *et al*. 1996a; 1996b; Vacelet 1996; Vacelet & Boury-Esnault 1996; Muricy *et al*. 1998; Vacelet & Perez 1998; Vacelet *et al*. 2000; Boury-Esnault 2002a; 2002b; Boury-Esnault & Bézac 2007; Bakran-Petricioli *et al*. 2007; Pisera & Vacelet 2011
Trypia Spilia (NA)	22-27	Current study
Grotta della Regina (AS)	22-25	Labate 1965
Leuca E. (IS)	22-24	Pulitzer-Finali & Pronzato 1981; Pulitzer-Finali 1983
Grotta Azzura (TS)	21	Sarà 1962; Benedetti-Cecchi *et al*. 1996a; 1996b; Southward *et al*. 1996; Benedetti-Cecchi *et al*. 1998
Piccola grotta ad W di Bonassola (LS)	20-23	Sarà 1964
Grotta delle Pecore (AS)	20-22	Sará 1961a
Monte Vico (Ischia) (TS)	20-21	Pulitzer-Finali 1970; Pulitzer-Finali & Pronzato 1977; Pulitzer-Finali 1978
J-1 (SC)	20-21	Bibiloni *et al*. 1989
Grotte de la Triperie (FC)	19-22	Vacelet 1964; Pouliquen 1969
Ftelio (NA)	18-20	Current study
J-2 (SC)	18-19	Bibiloni *et al*. 1989; Bibiloni 1993
Grotta dell'Arenile (AS)	17-18	Sará 1961a
Grotta delle Rondinelle (AS)	17-18	Sará 1961a
Columbera cave (AN)	15	Faresi *et al*. 2006
Secca delle Formiche di Vivara (TS)	14-15	Pulitzer-Finali 1970 ; Pulitzer-Finali & Pronzato 1977; Pulitzer-Finali 1978
Grotte del Coccodrillo	14-15	Sará 1961a

* Species richness is presented as a range, the lower value indicating the number of valid species and the higher including dubious species or taxa identified to the family or generic level, which imply hidden diversity.
