# Supplementary material for: Marine Caves of the Mediterranean Sea: A Sponge Biodiversity Reservoir within a Biodiversity Hotspot
Source: PLoS One. 2012 Jul 11;7(7):e39873. doi: 10.1371/journal.pone.0039873 (PMC3394755; doi:10.1371/journal.pone.0039873)
Supplement: Text S1 — Catalogue of literature on Mediterranean cave sponges. (PDF) [file pone.0039873.s005.pdf]

Text S1: Catalogue of literature on Mediterranean cave sponges.

Arillo A, Bavestrello G, Burlando B, Sarfi M (1993) Metabolic integration between symbiotic cyanobacteria and sponges: a possible mechanism. Mar Biol 117: 159-162.

Arko-Pijevac M, Benac Č, Kovačić M, Kirinčić M (2001) A submarine cave at the island of Krk (North Adriatic Sea). Nat Croatica 10: 163-184.

Bakran-Perticioli T, Radolović M, Petricioli D (2012) How diverse is sponge fauna in the Adriatic Sea? Zootaxa 3172: 20-38.

Bakran-Petricioli T, Vacelet J, Zibrowius H, Petricioli D, Chevaldonné P, et al. (2007) New data on the distribution of the 'deep-sea' sponges *Asbestopluma hypogea* and *Oopsacas minuta* in the Mediterranean Sea. Mar Ecol-Evol Persp 28: 10-23.

Balduzzi A, Bianchi CN, Boero F, Cattaneo R, Pansini M, et al. (1989) The suspension-feeder communities of a Mediterranean sea cave. Sci Mar 53: 387-395.

Balduzzi A, Pansini M, Pronzato R (1985) Estimation par relévements photographiques de la distribution de spongiaires et bryozoaires dans un grotte sous-marine du Golfe de Naples. Rapp Comm Int Mer Médit 29: 131-134.

Bavestrello G, Calcinai B, Cerrano C, Sarà M (1997) *Delectona madreporica* n. sp. (Porifera, Demospongiae) boring the corallites of some scleractinians from the Ligurian Sea. Ital J Zool 64: 273-277.

Belmonte G, Costantini A, Moscatello S, Denitto F, Shkurtaj B (2006) Le grotte sommerse della penisola del Karaburun (Albania): primi dati. Thalassia Salent 29: 15-28.

Ben Mustapha K, Komatsu K, Hattour A, Sammari C, Zarrouk S, et al. (2002) Tunisian megabenthos from infra (*Posidonia* meadows) and circalittoral (coralligenous) sites. Bull Inst Natl Sci Tech Mer (Salammbô) 29: 23-36.

Ben Mustapha K, Zarrouk A, Souissi A, El Abed A (2003) Diversité des demosponges tunisiennes. Bull Inst Natl Sci Tech Mer (Salammbô) 30: 55-77.

Benedetti-Cecchi L, Airoldi L, Abbiati M, Cinelli F (1996a) Estimating the abundance of benthic invertebrates: a comparison of procedures and variability between observers. Mar Ecol-Prog Ser 138: 93-101.

Benedetti-Cecchi L, Airoldi L, Abbiati M, Cinelli F (1996b) Exploring the causes of spatial variation in an assemblage of benthic invertebrates from a submarine cave with sulphur springs. J Exp Mar Biol Ecol 208: 153-168.

Benedetti-Cecchi L, Airoldi L, Abbiati M, Cinelli F (1998) Spatial variability in the distribution of sponges and cnidarians in a sublittoral marine cave with sulphur–water springs. J Mar Biol Ass UK 78: 43-58.

Bianchi CN, Cevasco MG, Diviacco G, Morri C (1986) Primi risultati di una ricerca ecologica sulla grotta marina di Bergeggi (Savona). Boll Mus Ist Biol Univ Genova 52: 267-293.

Bianchi CN, Morri C, Navone A (2010) I popolamenti delle scogliere rocciose sommerse dell'Area Marina Protetta di Tavolara Punta Coda Cavallo (Sardegna nord-orientale). Trav Sci Parc Nation Port-Cros 24: 39-86.

Bibiloni A, Gili JM (1982) Primera aportación al conocimiento de las cuevas submarinas de la isla de Mallorca. Oecol Aquat 6: 227-234.

Bibiloni A, Olivella I, Ros J (1984) Les esponges de les illes Medes. In: Ros J, Olivella I, Gilli JM, editors. Els sistemes naturals de les illes Medes. Barcelona: Arxius de la Seccio de Ciéncies. pp. 383-405.

Bibiloni MA (1993) Some new or poorly known sponges of the Balearic Islands (western Mediterranean). Sci Mar 57: 273-432.

Bibiloni MA, Gili JM, Ros JD (1984) Les coves submarines de les illes Medes. In: Ros J, Olivella I, Gilli JM, editors. Els sistemes naturals de les illes Medes. Institut d' Estudis Catalans: Arxius de la Seccio de Ciéncies. pp. 707-737.

Bibiloni MA, Uriz MJ, Gili JM (1989) Sponge communities in three submarine caves of the Balearic Islands (western Mediterranean): adaptations and faunistic composition. Mar Ecol-P S Z N I 10: 317-334.

Borg JA, Dimech M, Schembri PJ (2004) Report on a survey of the marine infralittoral benthic habitats in the Dwejra/Qawra area (Gozo, Maltese Islands). Mosta: Ecoserv. 30 p.

Boury-Esnault N (1971) Spongiaires de la zone rocheuse de Banyuls-sur-Mer. I. Ecologie et repartition. Vie Milieu B Oceanog 22: 159-192.

Boury-Esnault N (2002a) Family Polymastiidae Gray, 1867. In: Hooper JNA, van Soest RWM (2002) Systema Porifera. A guide to the classification of sponges. New York: Kluwer Academic/Plenum Publishers. pp. 201-219.

Boury-Esnault N (2002b) Order Chondrosida Boury-Esnault & Lopès, 1985. Family Chondrillidae Gray, 1872. In: Hooper JNA, van Soest RWM (2002) Systema Porifera. A guide to the classification of sponges. New York: Kluwer Academic/Plenum Publishers. pp. 291-297.

Boury-Esnault N, Bézac C (2007) Morphological and cytological descriptions of a new *Polymastia* species (Hadromerida, Demospongiae) from the North-West Mediterranean Sea. In: Custódio MR, Lôbo-Hajdu G, Hajdu E, Muricy G, editors. Porifera Research: Biodiversity, Innovation and Sustainability. Rio de Janeiro: Museu Nacional. pp. 23-30.

Boury-Esnault N, Muricy G, Gallissian MF, Vacelet J (1995) Sponges without skeleton: a new Mediterranean genus of Homoscleromorpha (Porifera, Demospongiae). Ophelia 43: 25-43.

Boury-Esnault N, Vacelet J (1994) Preliminary studies on the organization and development of a Hexactinellid sponge from a Mediterranean cave, *Oopsacas minuta*. In: van Soest RWM, van Kempen TMG, Braekman JC, editors. Sponges in time and space. Balkema Rotterdam. pp. 407-415.

Bussotti S, Terlizzi A, Fraschetti S, Belmonte G, Boero F (2006) Spatial and temporal variability of sessile benthos in shallow Mediterranean marine caves. Mar Ecol-Prog Ser 325: 109-119.

Carteron S (2002) Etude taxonomique des spongiaires du Liban. Stage de Maîtrise. Centre d'Océanologie de Marseille.

Cimino G, Crispino A, De Rosa S, De Stefano S, Sodano G (1981) Polyacetylenes from the sponge *Petrosia ficiformis* found in dark caves. Experientia 37: 924-926.

Cinelli F, Fresi E, Mazzella L, Pronzato M, Pansini M, et al. (1977) Distribution of benthic phyto- and zoocoenoses along a light gradient in a superficial marine cave. In: Keegan BF, O'Céidigh P, Boaden PJS, editors. Biology of benthic organisms. Oxford: Pergamon Press. pp 173-183.

Cocito S, Morganti C, Pansini M (2001) The sponge population of Tino and Tinetto Islands (Ligurian Sea): Distribution and abundance of the most conspicuous species (Porifera). Ann Mus Civ Stor Nat Giacomo Doria 94: 447-558.

Corriero G, Gherardi M, Giangrande A, Longo C, Mercurio M, et al. (2004) Inventory and distribution of hard bottom fauna from the Marine Protected area of Porto Cesareo (Ionian Sea): Porifera and Polychaeta. Ital J Zool 71: 237-245.

Corriero G, Liaci LS, Ruggiero D, Pansini M (2000) The sponge community of a semi-submerged Mediterranean cave. Mar Ecol-P S Z N I 21: 85-96.

Corriero G, Scalera Liaci L, Gristina M, Riggio S, Mercurio M (1997) Composizione tassonomica e distribuzione della fauna a poriferi e briozoi in una grotta

semisommersa della Riserva Naturale Marina "Isola di Ustica". Biol Mar Medit 4: 34-43.

Corriero G, Scalera Liaci L, Pronzato R (1996) Two new species of *Dendroxea* Griessinger (Porifera : Desmospongiae) from the Mediterranean Sea. Bull Inst R Sci Nat Belg 66: 197-203.

Corriero G, Scalera-Liaci L, Pronzato R (1997) *Didiscus spinoxeatus*, a new species of Porifera (Demospongiae) from the Mediterranean Sea. Ophelia 47: 63-70.

Deidun A, AIS Environmental Ltd (2006) Marine Scientific Surveys around Filfla for its conservation – draft management plan and monitoring report. Malta: Report compiled for AIS Environmental Ltd. 113 p.

Denitto F, Bussotti S, Costantini A, Poto M, Onorato R, et al. (2009) Prima indagine faunistica della grotta del Sifone (Canale d'Otranto, Salento meridionale, Italia). Thalassia Salent 32: 129-138.

Ereskovsky AV, Ivanisevic J, Pérez T (2009). Overview on the Homoscleromorpha sponges diversity in the Mediterranean. In: Proceedings of the First Mediterranean Symposium on the Coralligenous and other calcareous bio-concretions. Tunisia: Okianos. pp. 88-94.

Faresi L, Bettoso N, Aleffi IF (2006) Benthic macrofauna of a submarine cave on the Istrian Peninsula (Croatia). Annales Ser Hist Nat 16: 9-16.

Gili JM, Olivella I, Zabala M, Ros JD (1982) Primera contribución al conocimiento del poblamiento de las cuevas submarinas del litoral catalán. In: Niell FX, Ros JD, editors. Actas del Ier Simposio ibérico de Estudios del Bentos marino. San Sebastián. pp. 818-836.

Gràcia F, Clamor B, Jaume D, Fornós JJ, Uriz MJ, et al. (2005) La Cova des Coll (Felanitx, Mallorca): Espeleogènesi, geomorfologia, hidrologia, sedimentologia, fauna i conservació. Endins 27: 141-186.

Griessinger JM (1971) Étude des Réniérides de Méditerranée (Démosponges, Haplosclérides). Bull Mus Nation Hist Nat 3: 97-182.

Grubelic I, Antolic B, Span A (1998) Benthic flora and fauna in a submarine cave in the central Adriatic Sea. Rapp Comm Int Mer Médit 35: 446-447.

Harmelin JG, Boury-Esnault N, Fichez R, Vacelet J, Zibrowius H (2003) Peuplement de la grotte sous-marine de l'île de Bagaud (Parc national de Port-Cros, France, Méditerranée). Trav Sci Parc Nation Port-Cros 19: 117-134.

Jones DA, Knight-Jones EW, Moyse J, Babbage PC, Stebbing ARD (1968) Some biological problems in the Aegean. Underwater Ass Rep, Malta 1968: 73-78.

Labate M (1965) Ecologia dei Poriferi della grotta della Regina (Adriatico meridionale) Boll Zool 32: 541-553.

Laborel J (1960) Contribution a l'étude des peuplements benthiques sciaphiles sur substrat rocheux en Méditerranée. Rec Trav St Mar Endoume 33: 117-173.

Laborel J, Vacelet J (1958) Étude des peuplements d'une grotte sous-marine du Golfe de Marseille. Bull Inst Océanogr Monaco 1120: 1-20.

Laborel J, Vacelet J (1959) Les grottes sous-marines obscures en Méditerranée. CR Hebd Acad Sci 248: 2619-2621.

Manconi R, Ledda FD, Serusi A, Corso G, Stocchino GA (2009) Sponges of marine caves: Notes on the status of the Mediterranean palaeoendemic *Petrobiona massiliana* (Porifera: Calcarea: Lithonida) with new records from Sardinia. Ital J Zool 76: 306-315.

Manconi R, Serusi A (2008) Rare sponges from marine caves: discovery of *Neophrissospongia nana* nov sp (Demospongiae, Corallistidae) from Sardinia with an annotated checklist of Mediterranean lithistids. Zookeys 4: 71-87.

Manconi R, Serusi A, Pisera A (2006) A new Mediterranean 'lithistid' sponge, *Aciculites mediterranea* sp nov (Porifera : Demospongiae) from a dark marine cave in Sardinia. J Mar Biol Assoc UK 86: 691-698.

Martí R, Uriz MJ, Ballesteros E, Turón X (2004) Benthic assemblages in two Mediterranean caves: species diversity and coverage as a function of abiotic parameters and geographic distance. J Mar Biol Assoc UK 84: 557-572.

Martì R, Uriz MJ, Ballesteros E, Turon X (2004) Temporal variation of several structure descriptors in animal dominated benthic communities in two Mediterranean caves. J Mar Biol Assoc UK 84: 573-580.

Muricy G, Boury-Esnault N, Bézac C, Vacelet J (1996a) Cytological evidence for cryptic speciation in Mediterranean *Oscarella* species (Porifera, Homoscleromorpha). Can J Zoolog 74: 881-896.

Muricy G, Boury-Esnault N, Bézac C, Vacelet J (1998) Taxonomic revision of the Mediterranean *Plakina* Schulze (Porifera, Demospongiae, Homoscleromorpha). Zool J Linn Soc-Lond 124: 169-203.

Muricy G, Diaz MC (2002) Order Homosclerophorida Dendy, 1905. Family Plakinidae Schulze, 1880. In: Hooper JNA, van Soest RWM (2002) Systema Porifera. A guide to the classification of sponges. New York: Kluwer Academic/Plenum Publishers. pp. 71-82.

Muricy G, Solé-Cava AM, Thorpe JP, BouryEsnault N (1996b) Genetic evidence for extensive cryptic speciation in the subtidal sponge *Plakina trilopha* (Porifera: Demospongiae: Homoscleromorpha) from the Western Mediterranean. Mar Ecol-Prog Ser 138: 181-187.

Novosel M, Bakran-Petricioli T, Požar-Domac A, Kružić P, Radić I (2002) The benthos of the northern part of the Velebit Channel (Adriatic Sea, Croatia). Nat Croatica 11: 387-409.

Onorato R, Belmonte G, Costantini A (2006) Le grotte sommerse della costa neretina (Salento, S-E Italia). Thalassia Salent 29: 39-54.

Onorato R, Denitto F, Belmonte G (1999) Le grotte marine del Salento: classificazione, localizzazione e descrizione. Thalassia Salent 23: 67-116.

Onorato R, Forti P, Belmonte G, Costantini A, Poto M (2003) La grotta sottomarina lu Lampiùne: novità esplorative e prime indagini ecologiche. Thalassia Salent 26: 55-64.

Pansini M (1984) Notes on some Mediterranean *Axinella* with description of two new species. Boll Mus Ist Biol Univ Genova 50-51: 79-98.

Pansini M (1996) *Petrosia pulitzeri* n. sp. (Porifera, Demospongiae) from Mediterranean caves. Ital J Zool 63: 169-172.

Pansini M, Pesce GL (1998) *Higginsia ciccaresei* sp. nov. (Porifera: Demospongiae) from a marine cave on the Apulian coast (Mediterranean Sea). J Mar Biol Assoc UK 78: 1083-1091.

Pansini M, Pronzato R (1982). Distribuzione ed ecologia dei Poriferi nella grotta di Mitigliano (Penisola Sorrentina). Boll Mus Ist Biol Univ Genova 50: 287-293.

Pansini M, Pronzato R, Fresi E, Cinelli F, Mazzella L, et al. (1977) Evoluzione delle biocenosi bentoniche di substrato duro lungo un gradiente di luce in una grotta marina superficiale: Poriferi. In: Atti del IX Congresso della Società Italiana di Biologia Marina. Ischia. pp. 315-330.

Pérès JM, Picard J (1949) Notes sommaires sur le peuplement des grottes sous-marines de la région de Marseille. CR Soc Biogeogr 227: 42-45.

Pérez T, Ivanisevic J, Dubois M, Pedel L, Thomas OP, et al. (2011) *Oscarella balibaloi*, a new sponge species (Homoscleromorpha: Plakinidae) from the Western Mediterranean Sea: cytological description, reproductive cycle and ecology. Mar Ecol-Evol Persp 32: 174-187.

Pérez T, Vacelet J, Bitar G, Zibrowius H (2004) Two new lithistids (Porifera : Demospongiae) from a shallow eastern Mediterranean cave (Lebanon). J Mar Biol Assoc UK 84: 15-24.

Pisera A, Vacelet J (2011) Lithistid sponges from submarine caves in the Mediterranean: taxonomy and affinities. Sci Mar 75: 17-40.

Pouliquen L (1969) Remarques sur la présence d'éponges de l'étage bathyal dans les grottes sous-marines obscures en Méditerranée. CR Hebd Acad Sci 268: 1324-1326.

Pouliquen L (1972) Les spongiaires des grottes sous-marines de la région de Marseille. Ecologie et systématique. Téthys 3: 717-758.

Pulitzer-Finali G (1970) Report on a collection of sponges from the Bay of Naples. I. Sclerospongiae, Lithistida, Tetractinellida, Epipolasida. Pubbl Staz Zool Napoli 38: 328-354.

Pulitzer-Finali G (1978) Report on a collection of sponges from the Bay of Naples. III. Hadromerida, Axinellida, Poecilosclerida, Halichondrida, Haplosclerida. Boll Mus Ist Biol Univ Genova 45: 7-89.

Pulitzer-Finali G (1983) A collection of Mediterranean Demospongiae (Porifera) with, in appendix, a list of the Demospongiae hitherto recorded from the Mediterranean Sea. Ann Mus Civ Stor Nat Giacomo Doria, 84: 445-621.

Pulitzer-Finali G, Pronzato R (1977) Report on a collection of sponges from the Bay of Naples. II. Keratosa. Pubbl Staz Zool Napoli 40: 83-104.

Pulitzer-Finali G, Pronzato R (1981) The Keratosa in a collection of Mediterranean sponges mainly from the Italian coasts. Ann Mus Civ Stor Nat Giacomo Doria 83: 127-158.

Ramos-Esplá A, Cebrián D, Demetropoulos A (2007) Integrated coastal area management in Cyprus: biodiversity concerns on the coastal area management programme of Cyprus. Tunis: RAC/SPA. 69 p.

Reveillaud J, Remerie T, van Soest R, Erpenbeck D, Cárdenas P, et al. (2010). Species boundaries and phylogenetic relationships between Atlanto-Mediterranean shallow-water and deep-sea coral associated *Hexadella* species (Porifera, Ianthellidae). Mol Phylogenet Evol 56: 104-114.

Riedl R (1966) Biologie der Meereshöhlen. Hamburg: Paul Parey. 636 p.

Rosell D, Uriz MJ (2002) Excavating and endolithic sponge species (Porifera) from the Mediterranean: species descriptions and identification key. Org Diver Evol 2: 55-86.

Russ K, Rützler K (1959) Zur Kenntnis der Schwammfauna unterseeischer Höhlen. Pubbl Staz Zool Napoli 30: 756-787.

Rützler K (1965) Die Poriferen einer sorrentiner Höhle. Ergebnisse der Österreichischen Tyrrhenia Expedition 1952 Teil XVIII. Zool Anz 176: 303-319.

Rützler K (1965) Systematik und Ökologie der Poriferen aus Litoral-Schattengebieten der Nordadria. Z Morphol Ökol Tiere 55: 1-82.

Rützler K, Sarà M (1962) *Diplastrella ornata*, eine neue mediterrane Art der Familie Spirastrellidae (Demospongiae). Zool Anz 169: 231-236.

Sarà M (1958) Studio sui Poriferi di una grotta di marea del Golfo di Napoli. Arch Zool Ital 43: 203-281.

Sarà M (1959a) Considerazioni sulla distribuzione ed ecologia dei Poriferi nelle grotte. Ann Ist Mus Zool Napoli 11: 1-7.

Sarà M (1959b) Specie nuove di Demospongie provenienti da acque superficiali del golfo di Napoli. Ann Ist Mus Zool Napoli 11: 1-22.

Sarà M (1960a) Aspetti e problemi di una ricerca sinecologica sui Poriferi del Golfo di Napoli. Boll Zool 27: 51-65.

Sarà M (1960b) Poriferi del litorale dell' Ischia e loro ripartizione per ambienti. Pubbl Staz Zool Napoli 31: 421-472.

Sará M (1961a) La fauna di Poriferi delle grotte delle isole Tremiti. Studio ecologico e sistematico. Arch Zool Ital 46: 1-59.

Sará M (1961b) Zonazione dei poriferi nella grotta della "Gaiola". Ann Ist Mus Zool Napoli 13: 1-32.

Sarà M (1962a) Distribuzione ed ecologia dei Poriferi in acque superficiali del Golfo di Policastro (Mar Tirreno). Ann Inst Sci Lettere S Chiara 12: 191-214.

Sará M (1962b) Zonazione dei Poriferi in biotopi litorali. Pubbl Staz Zool Napoli 32: 44-57.

Sarà M (1964) Distribuzione ed ecologia dei Poriferi in acque superficiali della Riviera Ligure di Levante. Arch Zool Ital 49: 181-248.

Sará M (1968) Stratification des peuplements d'éponges à recouvrement total dans certaines grottes du niveau superficiel. Rapp Comm Int Mer Médit 19: 83-85.

Southward AJ, Kennicutt MC, Alcalà-Herrera J, Abbiati M, Airoldi L, et al. (1996) On the biology of submarine caves with sulphur springs: appraisal of 13C/12C ratios as a guide to trophic relations. J Mar Biol Assoc UK, 76: 265-285.

Steindler L, Schuster S, Ilan M, Avni A, Cerrano C, et al. (2007) Differential gene expression in a marine sponge in relation to its symbiotic state. Mar Biotechnol 9: 543-549.

Teixidó N, Garrabou J, Harmelin JG (2011) Low Dynamics, High Longevity and Persistence of Sessile Structural Species Dwelling on Mediterranean Coralligenous Outcrops. PLoS ONE 6: e23744.

True MA (1970) Étude quantitative de quatre peuplements sciaphiles sur substrat rocheux dansla région marseillaise. Bull Inst Oceanogr Monaco 69: 1-48.

Tsurnamal M (1969) Four new species of Mediterranean Demospongiae and new data on *Callites lacazei* Schmidt. Cah Biol Mar 10: 343-357.

Tsurnamal M (1975) The Calcareous sponges of shallow habitats along the Mediterranean coast of Israel. Israel J Zool 24: 137-153.

Turon X, Martí R, Uriz MJ (2009) Chemical bioactivity of sponges along an environmental gradient in a Mediterranean cave. Sci Mar 73: 387-397.

Uriz MJ, Rosell D, Martin D (1992) The sponge population of the Cabrera Archipelago (Balearic Islands): characteristics, distribution, and abundance of the most representative species. Mar Ecol-P S Z N I 13: 101-117.

Vacelet J (1959) Répartition générale des éponges et systématique des éponges cornées de la région de Marseille et de quelques stations méditerranéennes. Rec Trav St Mar Endoume 26: 39-101.

Vacelet J (1961) Quelques éponges remarquables de Méditerranée. Rev Trav Inst Pèches Marit 25: 351-354.

Vacelet J (1961) Spongiaires (Démosponges) de la région de Bonifacio (Corse). Rec Trav St Mar Endoume 22: 21-45.

Vacelet J (1964) Étude monographique de l'éponge calcaire pharétronide de Méditerranée, *Petrobiona massiliana* Vacelet et Lévi. Les Pharétronides actuelles et fossiles. Rec Trav St Mar Endoume 34: 1-125.

Vacelet J (1967) Quelques éponges Pharetronides et "Silico-Calcaires" de grottes sous-marines obscures. Rec Trav St Mar Endoume 42: 121-132.

Vacelet J (1976) Inventaire des Spongiaires du Parc National de Port-Cros (Var). Trav Sci Parc Nation Port-Cros 2: 167-186.

Vacelet J (1976) Les Spongiaires des grottes sous-marines obscures de la Méditerranée et des régions tropicales. Pubbl Staz Zool Napoli 40: 506-515.

Vacelet J (1996) Nouvelle signalisation d'éponges profondes en Méditerranée. Mésogée 55: 107-114.

Vacelet J (1999) Sponges (Porifera) in submarine caves. Qatar Univ Sci J 19: 46-56.

Vacelet J, Bitar G, Carteron S, Zibrowius H, Pérez T (2007) Five new sponge species (Porifera : Demospongiae) of subtropical or tropical affinities from the coast of Lebanon (eastern Mediterranean). J Mar Biol Assoc UK 87: 1539-1552.

Vacelet J, Borchiellini C, Perez T, Butel-Poncé V, Brouard JP, et al. (2000) Morphological, chemical and biochemical characterization of a new species of sponge without skeleton (Porifera, Demospongiae) from the Mediterranean Sea. Zoosystema 22: 313-326.

Vacelet J, Boury-Esnault N (1982) Une nouvelle éponge du genre *Crambe* (Demospongiae, Poecilosclerida) de Mediterranee, *C. tailliezi* n. sp. Trav Sci Parc Nation Port-Cros 8: 107-113.

Vacelet J, Boury-Esnault N (1996) A new species of carnivorous sponge (Demospongiae: Cladorhizidae) from a Mediterranean cave. Bull Inst R Sci Nat Belg Biol 66: 109-115.

Vacelet J, Boury-Esnault N, Harmelin JG (1994) Hexactinellid cave, a unique deep-sea habitat in the scuba zone. Deep-Sea Res Pt I 41: 965-973.

Vacelet J, Lévi C (1958) Un cas de survivance, en Méditerranée, du groupe d'éponges fossiles des Pharétronides. C CR Hebd Acad Sci 246: 318-320.

Vacelet J, Pérez T (1998) Two new genera and species of sponges (Porifera, Demospongiae) without skeleton from a Mediterranean cave. Zoosystema 20: 5-22.

van Lent F, de Weerdt WH (1987) The Haplosclerid sponge fauna of Banyuls-sur-mer (Mediterranean), with the description of a new species. In: Vacelet J, Boury-Esnault N, editors. Taxonomy of Porifera from the N.E. Atlantic and Mediterranean Sea. NATO ASI Series G13. pp. 125-148.

Vishnyakov AE, Ereskovsky AV (2009) Bacterial symbionts as an additional cytological marker for identification of sponges without a skeleton. Mar Biol 156: 1625-1632.

Voultsiadou E (1986) Systematics zoogeography and ecology of the demosponges (Porifera) of the continental shelf in the North Aegean Sea. Aristotle Unversity of Thessaloniki. 493 p.

Voultsiadou E (2005a) Demosponge distribution in the eastern Mediterranean: a NW-SE gradient. Helgoland Mar Res 59: 237-251.

Voultsiadou E (2005b) Sponge diversity in the Aegean Sea: check list and new information. Ital J Zool 72: 53-64.

Voultsiadou E, Vafidis D (2004) Rare sponge (Demospongiae, Porifera) from the Mediterranean Sea. J Mar Biol Assoc UK 84: 593-598.

Voultsiadou-Koukoura E, Koukouras A. (1993) Contribution to the knowledge of Keratose sponges (Dictyoceratida, Dendroceratida, Verongida: Demospongiae, Porifera) of the Aegean Sea. Mitt Zool Mus Berlin 69: 57-72.

Voultsiadou-Koukoura E, van Soest RWM (1991) *Hemiasterella aristoteliana* n. sp. (Porifera, Hadromerida) from the Aegean Sea with a discussion of the family Hemiasterellidae. Bijdr Dierk 61: 43-49.

Voultsiadou-Koukoura E, van Soest RWM, Koukouras A (1991) *Coscinoderma sporadense* sp. n. from the Aegean Sea with comments on *Coscinoderma confragosum* (Porifera, Dictyoceratida). Zool Scr 20: 195-199.

Zavodnik D, Pallaoro A, Jaklin A, Kovačić M, Arko-Pijevac M (2006) A benthos survey of the Senj Archipelago (North Adriatic Sea, Croatia). Acta Adriat 46: 3-68.
